# Thermal Stability of the Human Immunodeficiency Virus Type 1 (HIV-1) Receptors, CD4 and CXCR4, Reconstituted in Proteoliposomes

**DOI:** 10.1371/journal.pone.0013249

**Published:** 2010-10-13

**Authors:** Mikhail A. Zhukovsky, Stéphane Basmaciogullari, Beatriz Pacheco, Liping Wang, Navid Madani, Hillel Haim, Joseph Sodroski

**Affiliations:** 1 Department of Cancer Immunology and AIDS, Dana-Farber Cancer Institute, Department of Pathology, Division of AIDS, Harvard Medical School, Boston, Massachusetts, United States of America; 2 Department of Structural Dynamics of (Bio)chemical Systems, Max Planck Institute for Biophysical Chemistry, Göttingen, Germany; 3 Department of Immunology and Infectious Diseases, Harvard School of Public Health, Boston, Massachusetts, United States of America; Institut Pasteur, France

## Abstract

**Background:**

The entry of human immunodeficiency virus (HIV-1) into host cells involves the interaction of the viral exterior envelope glycoprotein, gp120, and receptors on the target cell. The HIV-1 receptors are CD4 and one of two chemokine receptors, CCR5 or CXCR4.

**Methodology/Principal Findings:**

We created proteoliposomes that contain CD4, the primary HIV-1 receptor, and one of the coreceptors, CXCR4. Antibodies against CD4 and CXCR4 specifically bound the proteoliposomes. CXCL12, the natural ligand for CXCR4, and the small-molecule CXCR4 antagonist, AMD3100, bound the proteoliposomes with affinities close to those associated with the binding of these molecules to cells expressing CXCR4 and CD4. The HIV-1 gp120 exterior envelope glycoprotein bound tightly to proteoliposomes expressing only CD4 and, in the presence of soluble CD4, bound weakly to proteoliposomes expressing only CXCR4. The thermal stability of CD4 and CXCR4 inserted into liposomes was examined. Thermal denaturation of CXCR4 followed second-order kinetics, with an activation energy (E_a_) of 269 kJ/mol (64.3 kcal/mol) and an inactivation temperature (T_i_) of 56°C. Thermal inactivation of CD4 exhibited a reaction order of 1.3, an E_a_ of 278 kJ/mol (66.5 kcal/mol), and a T_i_ of 52.2°C. The second-order denaturation kinetics of CXCR4 is unusual among G protein-coupled receptors, and may result from dimeric interactions between CXCR4 molecules.

**Conclusions/Significance:**

Our studies with proteoliposomes containing the native HIV-1 receptors allowed an examination of the binding of biologically important ligands and revealed the higher-order denaturation kinetics of these receptors. CD4/CXCR4-proteoliposomes may be useful for the study of virus-target cell interactions and for the identification of inhibitors.

## Introduction

Human immunodeficiency virus type 1 (HIV-1) entry into target cells is mediated by the viral envelope glycoproteins, following interaction with the host cell receptors, CD4 and one of two coreceptors, CCR5 or CXCR4 [1]–[3]. The HIV-1 envelope glycoproteins are organized into trimers consisting of three gp120 exterior envelope glycoproteins and three gp41 transmembrane envelope glycoproteins. The association of gp120 with gp41 in the trimer is maintained by non-covalent bonds. The unliganded HIV-1 envelope glycoproteins exist in a high-energy state. The binding of gp120 to CD4 results in envelope glycoprotein conformational changes that increase the affinity of gp120 for CCR5 or CXCR4. CD4 binding also results in an alteration of the gp120-gp41 relationship, leading to exposure of previously buried gp41 ectodomain segments. Subsequent binding of gp120 to CCR5 or CXCR4, which are members of the family of G protein-coupled, 7-transmembrane segment receptors, is thought to trigger additional conformational changes in the envelope glycoproteins. These changes may release constraints on the metastable gp41 glycoprotein. The formation of a stable six-helix bundle by the gp41 ectodomain is thought to drive the fusion of the viral and target cell membranes.

For primary HIV-1 isolates, CD4 and either CCR5 or CXCR4 are required for entry into the host cell. Most transmitted and early HIV-1 isolates use CCR5 as a coreceptor. In some HIV-1-infected individuals, the viruses acquire the ability to use CXCR4 as a coreceptor. Besides the presence of CD4 and the chemokine receptors, the lipid composition of the target cell membrane has also been suggested to influence the efficiency of virus-cell membrane fusion.

CD4 [4] is a Type 1 membrane protein consisting of four extracellular immunoglobulin-like domains (designated D1–D4), a transmembrane segment, and a cytoplasmic tail. The two amino-terminal CD4 domains (D1 and D2) contribute to the interaction with the natural CD4 ligand, the major histocompatibility complex class II (MHC II) protein, during the association of CD4-expressing T cells with antigen-presenting cells [5]. CD4 mainly exists as a 55-kDa monomer on cell surfaces, but can form weak dimers as a result of interactions involving domains D3 and D4 [6]. The cytoplasmic tail of CD4 is associated with a Src-family kinase, p56^lck^ [7], [8], and contributes to intracellular signaling in response to T-cell receptor triggering [9]. CD4 is used as a receptor by human and simian immunodeficiency viruses [10]. The viral gp120 glycoprotein binds CD4 domain D1 exclusively. The other CD4 domains are not absolutely required for HIV-1 entry, but contribute to the efficiency of the entry process, perhaps by orienting and spatially positioning D1.

CXCR4 [11]–[13] is a G protein-coupled receptor (GPCR) that acts as a receptor for the chemokine, CXCL12 (also known as stromal cell-derived factor (SDF-1)) [14], [15]. CXCR4 plays a role in fetal development, trafficking of naïve lymphocytes, mobilization of hematopoietic stem cells, migration of several types of neural cells, and synaptic transmission [16], [17]. CXCR4 has been implicated in different types of cancer [18]–[20], in the WHIM (Warts, Hypogammaglobulinemia, Infection and Myelokathexis) syndrome [21], [22], in rheumatoid arthritis [23], and in the immune response during fungal asthma [24]. CXCR4 is expressed on naïve T lymphocytes, which are much more abundant than the CCR5-expressing, activated T lymphocytes. Thus, the emergence of HIV-1 viruses that can utilize CXCR4 as a coreceptor typically exposes a much larger population of CD4-positive T lymphocytes to potential infection by HIV-1 and is usually associated with a poorer prognosis.

Proteoliposomes that contain the HIV-1 receptors could be useful tools in investigating the interaction of HIV-1 with target cells [25]. CD4-containing proteoliposomes have been produced [26]–[30] and shown to bind specifically to HIV-1-infected cells [26], [27]. Proteoliposomes containing CD4 and CCR5 also retain the ability to bind cells expressing HIV-1 envelope glycoproteins [31]. Paramagnetic proteoliposomes, consisting of a paramagnetic bead surrounded by a lipid membrane containing CCR5 [32] or CXCR4 [33], have been used to study the binding of natural chemokine ligands and HIV-1 gp120 glycoproteins. The gp120 glycoprotein of CCR5-using HIV-1 isolates was shown to bind specifically to CCR5-proteoliposomes in the absence of soluble CD4 (sCD4), but bound more efficiently in the presence of sCD4 [32]. The specific binding of gp120 glycoproteins from CXCR4-using HIV-1 variants to CXCR4-proteoliposomes was detected only after incubation with sCD4, and occurred with a dissociation constant of 200 nM [33]. Thus, the HIV-1 receptors can retain the ability to bind the HIV-1 gp120 envelope glycoprotein after solubilization, purification and reconstitution into proteoliposomes.

Here, we create proteoliposomes containing CD4 and CXCR4, demonstrate the ability of these HIV-1 receptors to interact with specific ligands, and investigate the thermal stability of these membrane proteins.

## Results

### Preparation of proteoliposomes containing CD4 and CXCR4

To create proteoliposomes, we used CHAPSO detergent to solubilize human CD4 (expressed in 293T cells) and human CXCR4 (expressed in Cf2Th cells). Both proteins have a C-terminal C9 epitope tag, allowing capture and purification with the 1D4 antibody complexed with Protein A-Sepharose CL-4B beads. After washing, the CD4 and CXCR4 proteins were eluted from the beads with the C12 peptide, which competes with the C9 epitope tag for the 1D4 antibody [34]. The eluted proteins were then incorporated into liposomes by dialysis against glycerol-free buffer in the presence of lipids. Proteoliposomes containing CD4, CXCR4 and both CD4 and CXCR4 were prepared. The proteoliposomes were freeze-thawed to enhance protein reconstitution in the membrane [35]–[37] and to decrease lamellarity [38]–[40], which can complicate interpretation of studies of proteoliposomes [41].

In some experiments, the proteoliposomes were extruded through polycarbonate filters. The non-extruded and extruded CXCR4-proteoliposomes were studied by dynamic light scattering and electron microscopy. The mean diameter of the non-extruded proteoliposomes was found to be approximately 2,000 nm and that of the extruded proteoliposomes around 200 nm (Figure S1).

The protein composition of the CD4-proteoliposomes and CXCR4-proteoliposomes was analyzed by separation on reducing and non-reducing SDS-polyacrylamide gels and silver staining. As a negative control, we prepared CD4-free and CXCR4-free liposomes from lysates of 293T cells and Cf2Th cells, respectively, and analyzed these in parallel. The 1D4 antibody could be detected in all of the liposomes (Figure S2). In addition, the CXCR4 protein was detected in the CXCR4-proteoliposomes and the CD4 protein in the CD4-proteoliposomes.

### Antibody binding to CD4/CXCR4-proteoliposomes

**Figure 1:**
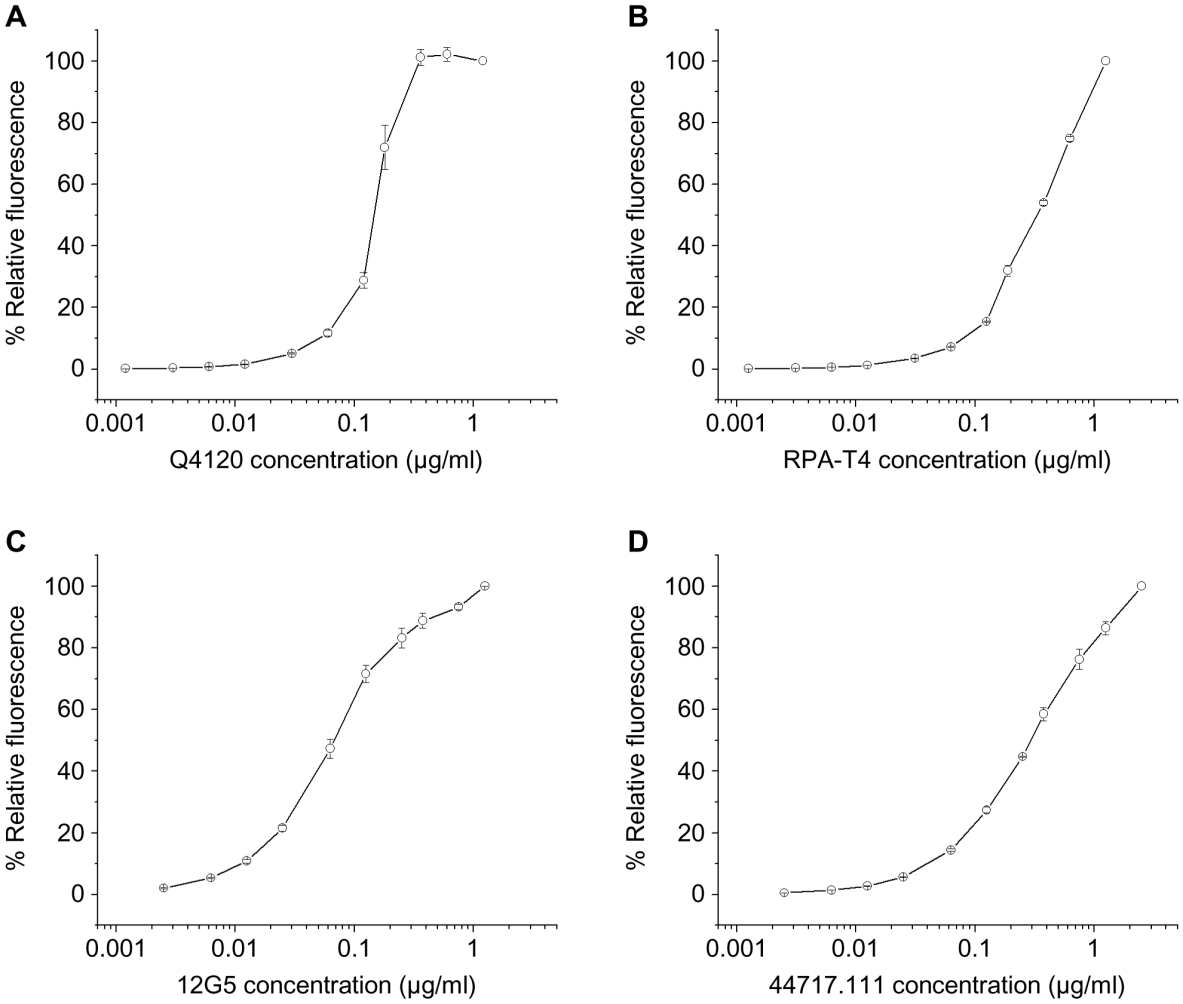
Binding of conformation-dependent monoclonal antibodies against CD4 and CXCR4 to CD4/CXCR4-proteoliposomes. The indicated concentrations of PE-labeled monoclonal antibodies Q4120 (**A**), RPA-T4 (**B**), 12G5 (**C**) and 44717.111 (**D**) were incubated with CD4/CXCR4-proteoliposomes. The mean fluorescence intensity at each antibody concentration was normalized to that seen at the highest antibody concentration used. Each data point represents the mean and standard error derived from three independent experiments, each using a different preparation of CD4/CXCR4-proteoliposomes.

To assess the conformation of the CD4 and CXCR4 molecules incorporated into the proteoliposomes, we studied the binding of phycoerythrin (PE)-labeled antibodies to CD4/CXCR4-proteoliposomes using flow cytometry. Two anti-CD4 antibodies, Q4120 [42] and RPA-T4 [43], were used. The Q4120 antibody recognizes an epitope in the D1 domain of CD4 that is sensitive to denaturation [44]. The RPA-T4 antibody binds to a D1 epitope that involves the complementarity-determining region (CDR) loops 1 and 3. Both antibodies bound to CD4/CXCR4-proteoliposomes in a dose-dependent manner (Figure 1).

We also tested two antibodies, 12G5 [45] and 44717.111 [46], that are directed against conformation-dependent epitopes on CXCR4. The 12G5 antibody recognizes only a subset of CXCR4 conformations [46], [47] and binds a complex epitope influenced by the integrity of the CXCR4 second extracellular loop [47], [48] and the disulfide bond between cysteine residues 28 and 274 [47]. The 44717.111 antibody reacts preferentially with the second extracellular loop of CXCR4 and is capable of binding multiple CXCR4 conformations [46], [47]. Both antibodies bound to CD4/CXCR4-proteoliposomes in a dose-dependent manner (Figure 1). The entire population of CD4/CXCR4-proteoliposomes stained positively with an anti-CD4 and an anti-CXCR4 antibody, indicating that essentially all of the proteoliposomes contain both proteins. A control antibody, 2D7 [49], which is directed against CCR5, did not bind the CD4/CXCR4-proteoliposomes efficiently (data not shown). Moreover, none of the anti-CD4 or anti-CXCR4 antibodies bound liposomes prepared without CD4 and CXCR4. These results suggest that both CD4 and CXCR4 in the proteoliposomes were in native conformations. The 12G5 and 44717.111 antibodies also bound to the extruded CXCR4-proteoliposomes (data not shown), indicating that CXCR4 can be individually reconstituted into extruded proteoliposomes in a native state.

Several of the variables associated with the preparation of CD4/CXCR4-proteoliposomes were optimized by assessing the final product with the Q4120 anti-CD4 antibody and the 12G5 anti-CXCR4 antibody. These studies revealed that the use of glycerol-free dialysis buffer resulted in a better yield of proteoliposomes with conformationally correct CD4 and CXCR4 (data not shown). The concentration of 1D4 antibody used to capture CD4 and CXCR4 (1.1 mg/ml) and the concentration of C12 peptide used to elute these proteins from the beads (200 µg/ml) were determined by optimizing 12G5 antibody recognition. In a similar manner, elution of CD4 and CXCR4 from the beads at room temperature proved to be slightly better than elution at 4°C for producing optimal levels of CD4 and CXCR4 recognized by the conformation-dependent antibodies (data not shown). Recognition by conformation-dependent anti-CD4 and anti-CXCR4 antibodies was maintained for CD4/CXCR4-proteoliposomes stored at 4°C for at least 3–4 weeks, with an affinity comparable to that seen for freshly prepared proteoliposomes (data not shown).

### Binding of a small molecule to CD4/CXCR4-proteoliposomes and cells

**Figure 2:**
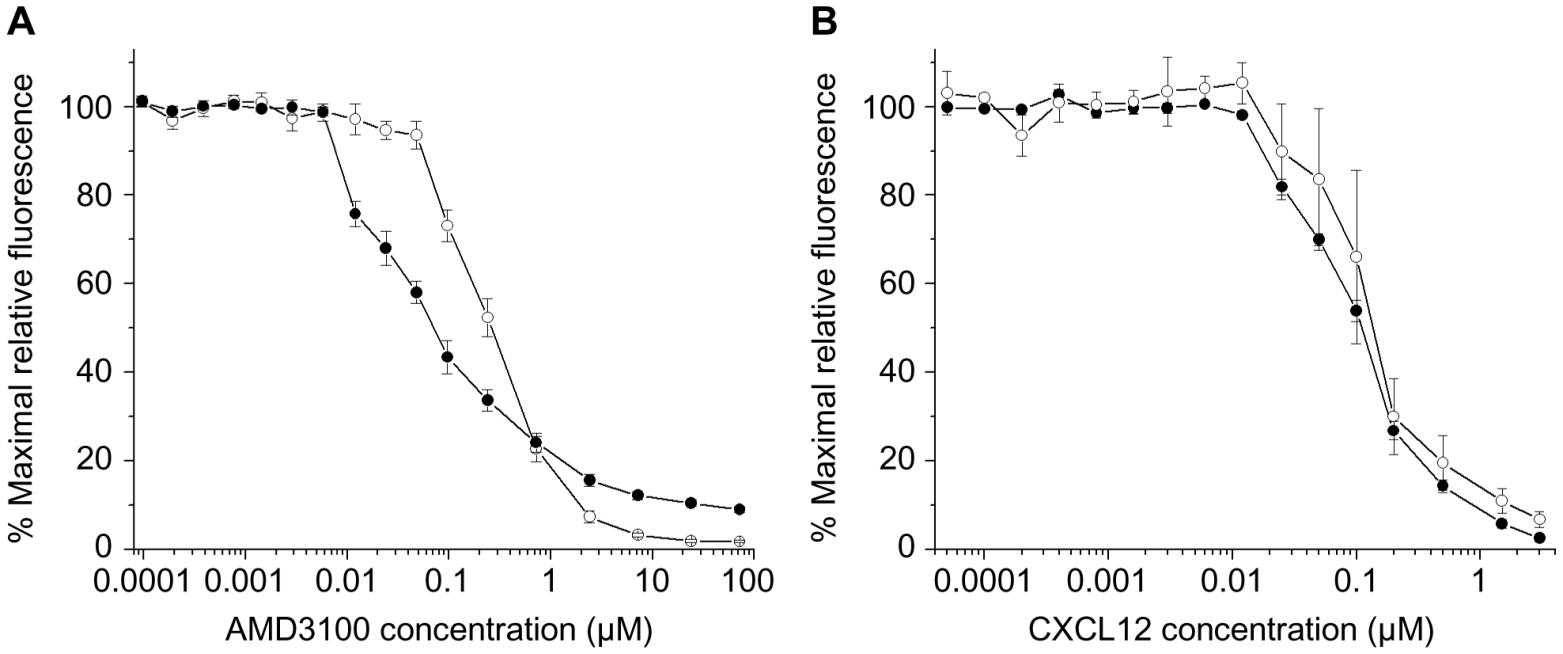
Binding of AMD3100 and CXCL12 to CD4/CXCR4-proteoliposomes and CD4/CXCR4-expressing cells. The indicated concentrations of AMD3100 (**A**) or CXCL12 (**B**) were added to CD4/CXCR4-proteoliposomes (filled circles) or Cf2Th-CD4/CXCR4 cells (open circles) in the presence of 1.25 µg/ml (**A**) or 0.25 µg/ml (**B**) PE-labeled anti-CXCR4 antibody 12G5. The mean fluorescence intensity at each concentration of AMD3100 or CXCL12 was normalized to the maximum value observed in the series. Each data point represents the mean and standard error derived from three independent experiments. Each of the three experiments with CD4/CXCR4-proteoliposomes used a different preparation of CD4/CXCR4-proteoliposomes.

The bicyclam AMD3100 [50]–[53] is a small molecule (molecular weight 830 Da) that specifically binds CXCR4 and inhibits the infection of HIV-1 isolates that utilize CXCR4 as the receptor [54]. The ability of AMD3100 to compete [46], [55] with the binding of PE-labeled 12G5 anti-CXCR4 antibody to CD4/CXCR4-proteoliposomes and cells expressing CD4 and CXCR4 was examined. At a 12G5 concentration of 1.25 µg/ml, AMD3100 competed for binding of the antibody to CXCR4-expressing Cf2Th cells with a half-maximal inhibitory concentration (IC_50_) of 270 nM (Figure 2A). Of interest, AMD3100 exhibited an IC_50_ of 70 nM when the 12G5 competition was performed with CD4/CXCR4-proteoliposomes. This suggests that AMD3100 binds better to CXCR4 on the surface of the proteoliposomes than on the cell surface. The inhibition of 12G5 binding by AMD3100 was not as complete for the CD4/CXCR4-proteoliposomes as for the CXCR4-expressing cells, suggesting differences in binding cooperativity or CXCR4 conformational states between the proteoliposomes and cells. The observed inhibition was specific, as AMD3100 did not inhibit the binding of the Q4120 and RPA-T4 anti-CD4 antibodies either to CD4/CXCR4-proteoliposomes (Table S1) or to Cf2Th-CD4/CXCR4 cells (data not shown). AMD3100 also inhibited 12G5 binding to extruded CXCR4-proteoliposomes (data not shown). CCR5 antagonists, TAK779 [56] and Compound A [57], did not inhibit 12G5 binding either to CD4/CXCR4-proteoliposomes (Table S1) or to Cf2Th-CD4/CXCR4 cells (data not shown). We conclude that AMD3100 binds efficiently to CXCR4 on the CD4/CXCR4-proteoliposomes.

### CXCL12 binding to CD4/CXCR4-proteoliposomes and cells

We examined the binding of the natural CXCR4 ligand, CXCL12 [14], [15], [58], [59], to CD4/CXCR4-proteoliposomes. The shorter, 68-residue α isoform [60] of CXCL12 was used for these studies. CXCL12 competed [46], [55] with phycoerythrin (PE)-labeled 12G5 antibody for binding to CXCR4. The ability of increasing concentrations of CXCL12 to compete for 12G5 binding to CD4/CXCR4-proteoliposomes was compared with the ability to compete for 12G5 binding to Cf2Th cells expressing CD4 and CXCR4. The observed inhibitory curves were almost identical (Figure 2B). When the concentration of the 12G5 antibody was 0.25 µg/ml, the half-maximal inhibitory concentration (IC_50_) of CXCL12 was 110 nM for CD4/CXCR4-proteoliposomes and 140 nM for CD4/CXCR4-expressing cells.

CXCL12 also inhibited the binding of the 44717.111 anti-CXCR4 antibody to the CD4/CXCR4-proteoliposomes, but did not compete with the binding of the Q4120 and RPA-T4 anti-CD4 antibodies to the proteoliposomes (data not shown). These data suggested that the CXCR4 protein in the CD4/CXCR4-proteoliposomes binds CXCL12 with an affinity similar to that of CXCR4 expressed on the surface of cells.

### Binding of HIV-1 gp120 to CD4-proteoliposomes and CXCR4-proteoliposomes

**Figure 3:**
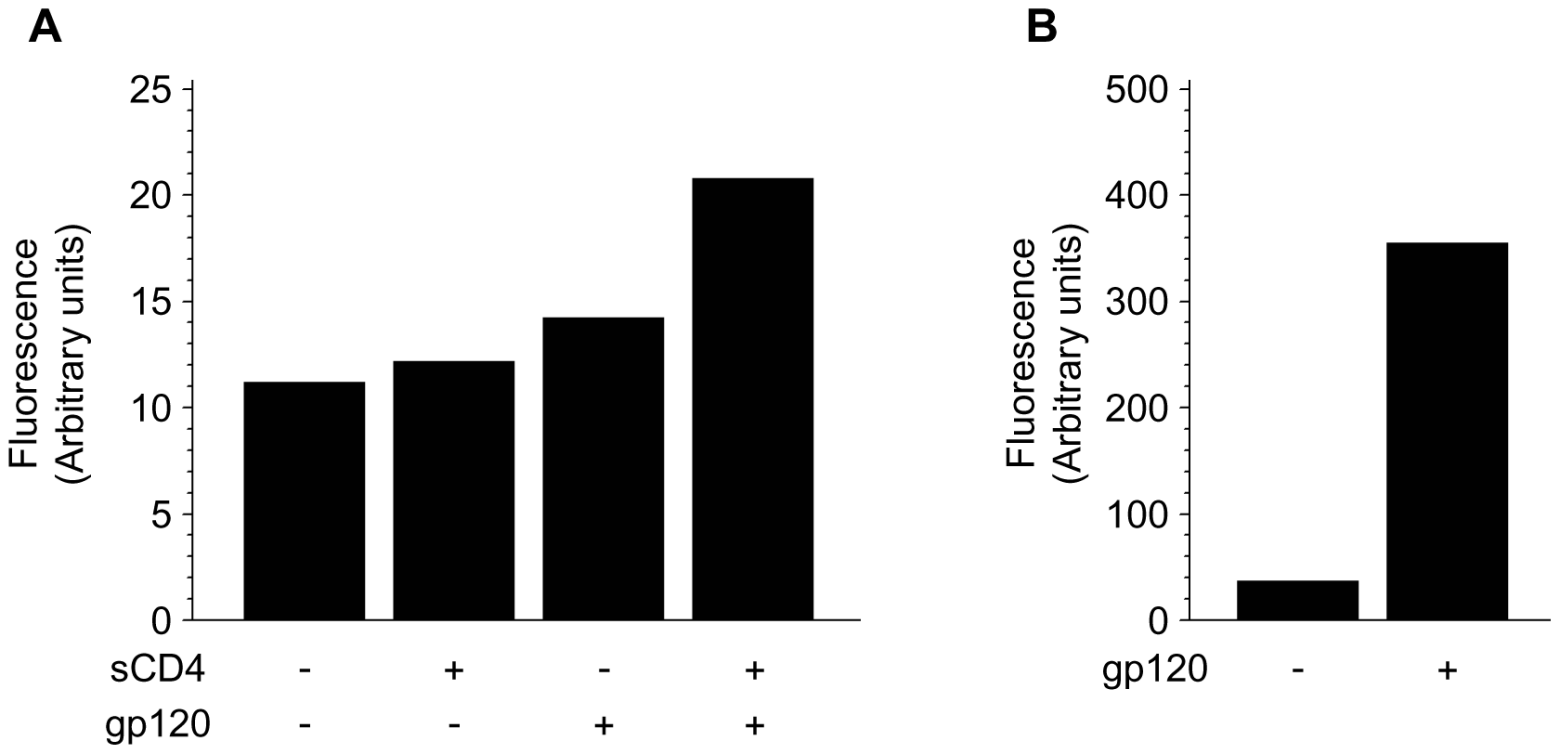
Binding of HIV-1 gp120 to CXCR4-proteoliposomes and CD4-proteoliposomes. The binding of HIV-1_HXBc2_ gp120 to CXCR4-proteoliposomes (**A**) or CD4-proteoliposomes (**B**) is shown. In some of the experiments shown in (**A**), soluble CD4 (sCD4) was added at a concentration of 80 µg/ml. The bound gp120 was detected by incubation of the proteoliposomes with the 2G12 anti-gp120 monoclonal antibody, followed by a PE-labeled goat anti-human antibody. The values shown represent the averages of the mean fluorescence intensities from 2–4 experiments.

FACS analysis was used to assess the binding of gp120 from the CXCR4-using HIV-1_HXBc2_ strain to CXCR4-proteoliposomes, in either the presence or the absence of sCD4. The bound gp120 molecules were detected with the human anti-gp120 antibody 2G12, followed by staining with a PE-labeled anti-human antibody. Only in the presence of sCD4 was weak binding of gp120 to the CXCR4-proteoliposomes detected above the background levels of fluorescence observed in untreated cells or cells incubated only with sCD4 (Figure 3A). These results are consistent with the previously reported [33] low affinity of HIV-1 gp120 for CXCR4, even in the presence of sCD4.

By contrast, strong specific binding of gp120 to CD4-proteoliposomes was detected (Figure 3B). The resulting mean fluorescence intensity was approximately 16-fold greater than that observed for gp120 binding to CXCR4-proteoliposomes in the presence of sCD4. This is consistent with the significantly greater affinity of gp120 for CD4 than for CXCR4.

### Fusogenicity of proteoliposomes

Previous studies have suggested that the multilamellar nature of liposomes can decrease their ability to participate in membrane fusion events [61], [62]. We evaluated the fusogenicity of extruded and non-extruded proteoliposomes by a lipid mixing assay. Polyethylene glycol (PEG)-mediated lipid mixing between proteoliposomes and cells was studied using flow cytometry. Proteoliposomes were synthesized in the presence of high concentrations of rhodamine-labeled lipid; this results in self-quenching of the dye in the lipid bilayer of the liposome. Should fusion occur with rhodamine-free liposomes or cells, lipid mixing would lead to rhodamine dilution, decreased quenching, and a net increase in rhodamine fluorescence [63], [64]. Both extruded and non-extruded CXCR4-proteoliposomes participated in PEG-mediated fusion with 293T cells (Figure S3).

We next analyzed the ability of extruded proteoliposomes containing the HIV-1 receptors to engage in a fusion reaction with 293T cells expressing the dual-tropic (CXCR4/CCR5-using) HIV-1_KB9_ glycoproteins, or with control 293T cells transfected with a plasmid containing the deleted ΔKS env gene. The expression of the HIV-1 envelope glycoproteins on the surface of 293T cells transfected with the plasmid containing the intact HIV-1 env gene, but not the deleted ΔKS env gene, was confirmed by staining with the 2G12 anti-gp120 antibody (data not shown). These 293T cells were incubated with either CD4-proteoliposomes or CD4/CXCR4-proteoliposomes. The ΔKS-transfected or HIV-1_KB9_ envelope-expressing 293T cells demonstrated a similar level of fluorescence following incubation with rhodamine-labeled CD4-proteoliposomes. Incubation of CD4/CXCR4-proteoliposomes with ΔKS-transfected cells resulted in a higher background level of fluorescence than was observed for the CD4-proteoliposomes; the basis for this background is unknown. The fluorescence increase associated with the incubation of CD4/CXCR4-proteoliposomes with 293T cells expressing the HIV-1_KB9_ envelope glycoproteins was slightly greater than that observed with the control ΔKS-transfected 293T cells (Figure S4). These results are consistent with a low level of HIV-1 envelope glycoprotein-dependent fusion occurring between the envelope-expressing 293T cells and the CD4/CXCR4-proteoliposomes.

### Thermal denaturation of CXCR4

Because proteoliposomes can withstand a wider range of temperatures than living cells, the availability of proteoliposomes containing the HIV-1 receptors provided an opportunity to examine the effects of temperature on the native conformation of these membrane proteins. The binding of a conformation-dependent ligand has been used to assess the degree of denaturation of some GPCRs following heat treatment [65]–[68]. Recognition of CXCR4 by four different conformation-dependent antibodies, as well as by CXCL12 and HIV-1 gp120, has been shown to be equivalently sensitive to detergent denaturation of CXCR4 [33], [69], [70]. To study the heat inactivation of CXCR4, we chose the conformation-dependent 12G5 anti-CXCR4 antibody [45]. We examined the interaction, at room temperature, of the PE-labeled 12G5 antibody with CD4/CXCR4-proteoliposomes that had been incubated at different temperatures for various times. The fluorescence observed for CD4/CXCR4-proteoliposomes incubated in parallel and then stained with a PE-labeled anti-CCR5 antibody, 2D7, was subtracted from the values observed with the 12G5 antibody.

**Figure 4:**
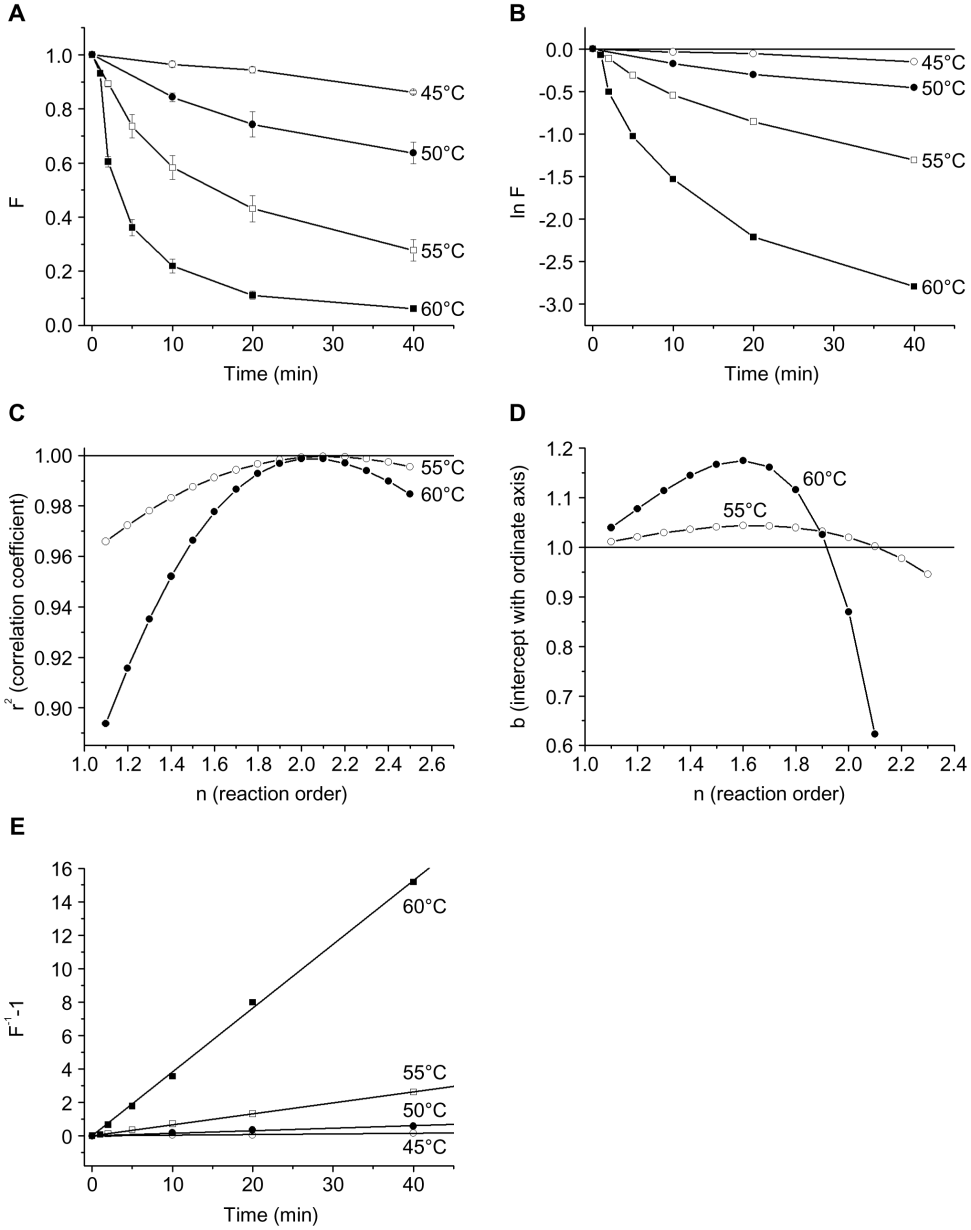
Temperature stability of CXCR4 in CD4/CXCR4-proteoliposomes. (**A**) The fluorescent staining of CD4/CXCR4-proteoliposomes with the PE-labeled anti-CXCR4 antibody 12G5 after incubation for the indicated time at the given temperature is shown. The F value represents the fluorescent staining relative to that observed in the absence of heat treatment of the CD4/CXCR4-proteoliposomes. Each data point represents the mean and standard error derived from three independent experiments, in which three different proteoliposome preparations were used. (**B**) The natural logarithm of F is shown as a function of time of incubation at the indicated temperatures. Note the non-linearity of the curves. (**C**) The correlation coefficient, r^2^, of the function F^1−n^ is shown for the fitting of the experimental data on CXCR4 thermal denaturation at 55°C and 60°C to equation (5) for the indicated reaction order n. (**D**) The intercept with the ordinate axis b of the function F^1−n^ is shown for the fitting of the experimental data on CXCR4 thermal denaturation at 55°C and 60°C to equation (5) for the indicated reaction order n. (**E**) The value F^−1^−1 as a function of the time of incubation of CD4/CXCR4-proteoliposomes at the indicated temperature is shown.

Incubation at higher temperatures resulted in progressively greater denaturation of CXCR4 (Figure 4A). When the natural logarithm of the remaining fluorescent staining by the 12G5 antibody (F) was plotted versus time, the non-linearity of the curves indicated that the thermal denaturation of CXCR4 did not follow first-order kinetics (Figure 4B). To determine the order of the thermal denaturation reaction, F^1−n^ at 55°C and 60°C (where sufficient denaturation occurred to make the measurements reliable) was fitted to equation (5) (See Materials and Methods). The correlation coefficient (r^2^) and the ordinate intercept (b) were close to 1 when the reaction order, n, was 2 (Figures 4C and 4D). Thus, the thermal denaturation of CXCR4 is a second-order reaction and equation (5) can be written as:
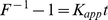
where t is time and K_app_ is the apparent reaction rate.

The F^−1^−1 versus time plots (Figure 4E) revealed the expected straight lines; the apparent reaction rates (K_app_) at each temperature were derived from the slopes of these lines (Table 1).

**Table 1:** Parameters of the thermal denaturation of CXCR4 and CD4 incorporated in CD4/CXCR4-proteoliposomes.

	CXCR4			CD4		
T(°C)	K_app_ (1/min)	Half-life (min)	D value (min)	K_app_ (1/min)	Half-life (min)	D value (min)
**45**	0.0038	263	2368	0.0086	89.6	386
**50**	0.0151	66.4	598	0.0342	22.5	97
**55**	0.0658	15.2	137	0.1923	4.01	17.3
**60**	0.3813	2.62	23.6	0.9385	0.82	3.54

The half-lives (t_1/2_) and decimal reduction time (D) (time required for 90% reduction in 12G5 antibody binding) were obtained for the second-order thermal denaturation of CXCR4 at each temperature from equations (7) and (9) (See Materials and Methods):
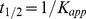


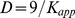



The calculated t_1/2_ and D values for CXCR4 at each temperature are shown in Table 1.

**Figure 5:**
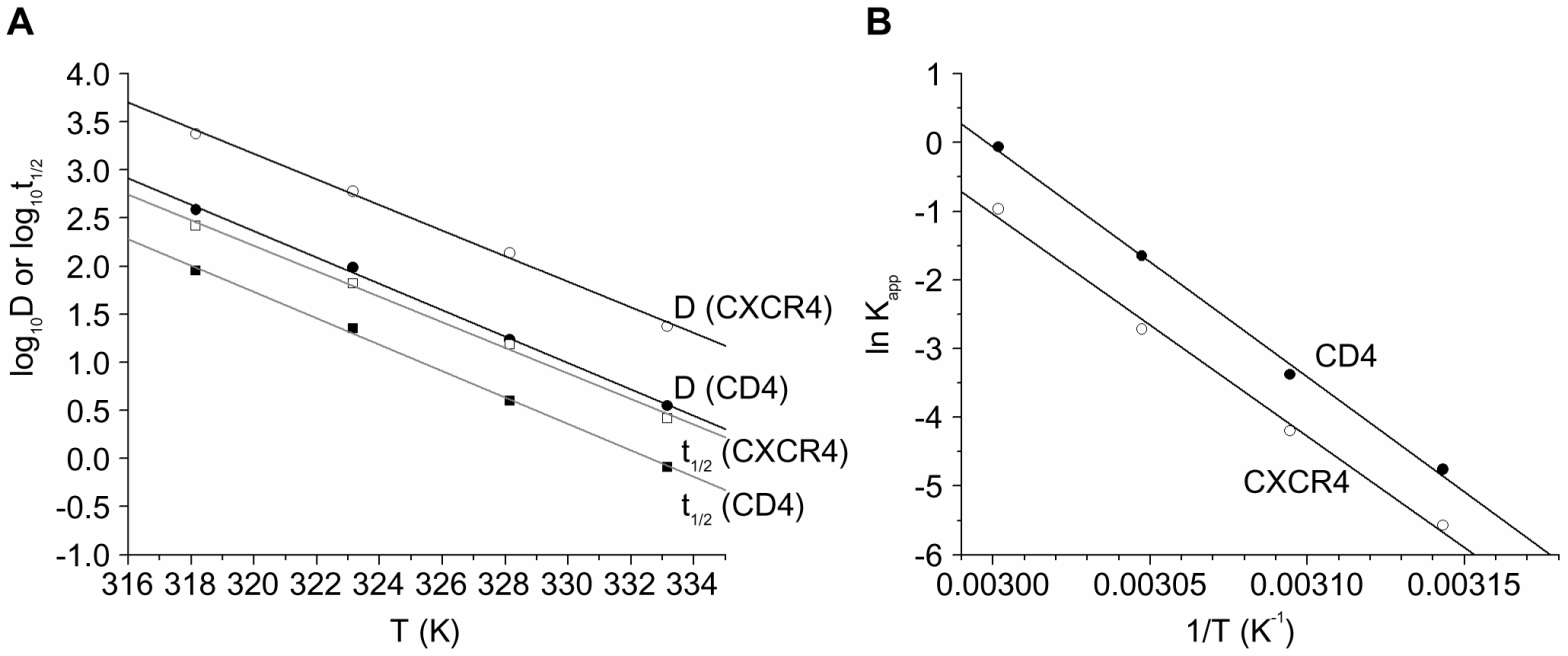
Activation energies and Z values for the thermal denaturation of CD4 and CXCR4 in CD4/CXCR4-proteoliposomes. (**A**) The decimal logarithm of the D value and half-life (t_1/2_) of native CD4 and CXCR4 in the CD4/CXCR4-proteoliposomes is shown as function of the absolute temperature. The Z values were derived from the slopes of the plots for log_10_D, and the T_i_ values from the slopes and ordinate intercepts of the plots for log_10_t_1/2_. (**B**) An Arrhenius plot shows the relationship of ln K_app_ and the reciprocal of the absolute temperature for CD4 and CXCR4 denaturation in the CD4/CXCR4-proteoliposomes. The slopes of these plots were used to determine the E_a_ values.

The Z value, a measure of the temperature dependence of the denaturation reaction rate, can be determined from a plot of log_10_D versus temperature (Figure 5A). The calculated Z value for CXCR4 is 7.5°C.

The inactivation temperature (T_i_), which is defined as the temperature at which the t_1/2_ of thermal denaturation is 10 minutes, can be found from a plot of log_10_t_1/2_ versus temperature (Figure 5A). The inactivation temperature for CXCR4 is 56.0°C.

The activation energy (E_a_) of the denaturation reaction (the minimal energy required to initiate the reaction) can be determined from a plot of ln K_app_ versus reciprocal temperature (Figure 5B). The activation energy for the thermal denaturation of CXCR4 is 269 kJ/mol, or 64.3 kcal/mol.

### Thermal denaturation of CD4

The thermal inactivation of CD4 in the CD4/CXCR4-proteoliposomes was studied by examining the binding, at room temperature, of the PE-labeled anti-CD4 antibody Q4120 to the proteoliposomes that had been preincubated at different temperatures for various times. The fluorescence observed with the anti-CCR5 antibody 2D7 was subtracted from the values observed with the Q4120 antibody.

**Figure 6:**
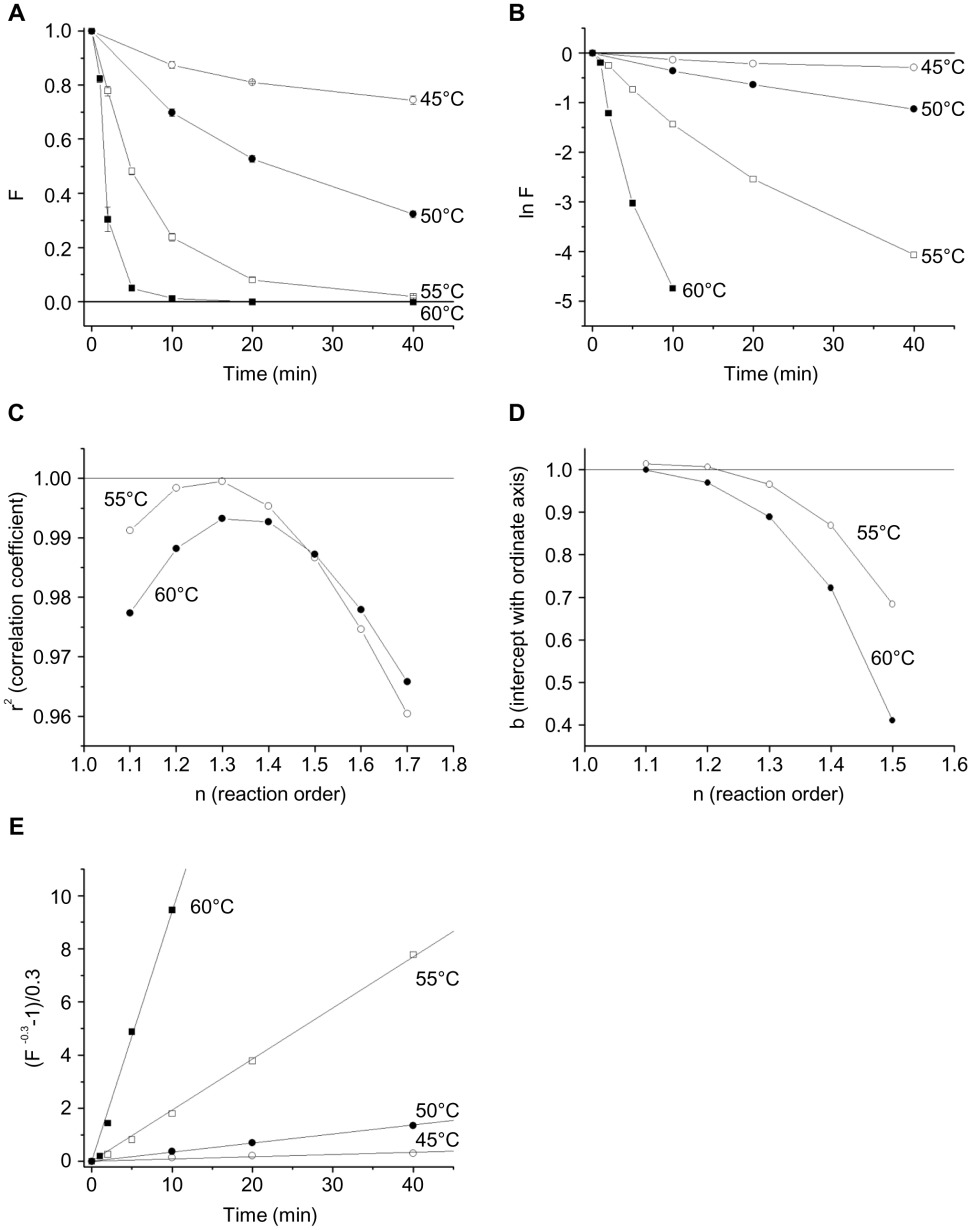
Temperature stability of CD4 in CD4/CXCR4-proteoliposomes. (**A**) The fluorescent staining of CD4/CXCR4-proteoliposomes with the PE-labeled anti-CD4 antibody Q4120 after incubation for the indicated time at the given temperature is shown. The F value represents the fluorescent staining relative to that observed in the absence of heat treatment of the CD4/CXCR4-proteoliposomes. Each data point represents the mean and standard error derived from three independent experiments, in which three different proteoliposome preparations were used. (**B**) The natural logarithm of F is shown as a function of time of incubation at the indicated temperatures. Note the non-linearity of the curves. (**C**) The correlation coefficient, r^2^, of the function F^1−n^ is shown for the fitting of the experimental data on CD4 thermal denaturation at 55°C and 60°C to equation (5) for the indicated reaction order n. (**D**) The intercept with the ordinate axis b of the function F^1−n^ is shown for the fitting of the experimental data on CD4 thermal denaturation at 55°C and 60°C to equation (5) for the indicated reaction order n. (**E**) The value (F^−0.3^−1)/0.3 as a function of the time of incubation of CD4/CXCR4-proteoliposomes at the indicated temperature is shown.

CD4 staining by the Q4120 antibody decreased faster than CXCR4 staining by the 12G5 antibody (compare Figures 4A and 6A). Because the plots of ln F versus time were not linear (Figure 6B), the thermal denaturation of CD4, like that of CXCR4, apparently does not follow first-order kinetics. Fitting of F^1−n^ to equation (5) at 55°C and 60°C suggested that the order (n) of the CD4 thermal denaturation is close to 1.3 (Figure 6C). The determination of the reaction order using the ordinate intercept b yields similar values (n = 1.2 for 55°C and n = 1.1 for 60°C) (Figure 6D). For n = 1.3, equation (5) can be written:
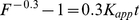
and the apparent reaction rate (K_app_) at each temperature can be calculated, using the slopes of the plots in Figure 6E.

Using equations (7) and (9) respectively, the t_1/2_ and D values at each temperature can be calculated:
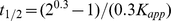


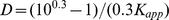
The values of K_app_, t_1/2_ and D are presented in Table 1.

From a plot of log_10_D versus temperature (Figure 5A), the value of Z was calculated to be 7.3°C. From the plot of log_10_t_1/2_ versus temperature (Figure 5A), the value for T_i_ was calculated to be 52.2°C.

The activation energy E_a_ of 278 kJ/mol = 66.5 kcal/mol of the CD4 denaturation reaction was derived from a plot of ln K_app_ versus reciprocal temperature (Figure 5B).

## Discussion

Proteoliposomes containing HIV-1 coreceptors, individually or together with CD4, have been reported [31]–[33]. Here we produced proteoliposomes containing CD4 and CXCR4. The CD4 and CXCR4 proteins in the proteoliposomes are in native conformations, based on recognition by HIV-1 gp120 and monoclonal antibodies. Moreover, CXCR4 in the proteoliposomes binds CXCL12, the natural ligand, and AMD3100, a small-molecule inhibitor of HIV-1 infection. Binding of CXCL12 to CXCR4, for example, involves interaction of the chemokine with the N terminus and extracellular loops 2 and 3 of CXCR4 [58], [60], supporting the assertion that CXCR4 in the proteoliposomes is in a native conformation. Unlike paramagnetic proteoliposomes used previously, the CD4/CXCR4-proteoliposomes studied here presumably have CD4 and CXCR4 molecules randomly oriented in either direction in the membrane; however, unlike the case for paramagnetic proteoliposomes, CD4 and CXCR4 in our proteoliposomes are not constrained with respect to lateral movement in the membrane. The lateral mobility may be helpful in allowing more natural clustering of receptor molecules bound to the HIV-1 envelope glycoprotein complex [70]–[73].

The specific interaction of the extruded CD4/CXCR4-proteoliposomes with cells expressing the HIV-1 envelope glycoproteins was studied. However, the background fluorescence signal associated with the interaction of the CD4/CXCR4-proteoliposomes with control cells not expressing envelope glycoproteins was significantly higher than that of CD4-proteoliposomes. The basis for this high background advises caution in interpreting the higher fluorescence associated with the interaction of CD4/CXCR4-proteoliposomes and cells expressing the HIV-1 envelope glycoproteins. Although some of the observed increase in fluorescence may result from bona fide membrane fusion, further work will be required to increase the efficiency of this process. For example, it has been suggested that glycosphingolipids in the target membrane play an important role in membrane fusion mediated by the HIV-1 envelope glycoproteins [74]–[79]. Future experiments will address whether CD4/CXCR4-proteoliposomes prepared with modified lipid compositions, including glycosphingolipids, will act as more efficient targets for HIV-1 envelope glycoprotein-mediated membrane fusion.

The thermal stability of the HIV-1 receptors has not been studied in detail. The integrity of the conformation-dependent CCR5 epitope recognized by the 2D7 antibody was preserved after exposure of CCR5-paramagnetic proteoliposomes to 50°C [32], but the denaturation of CCR5 in this context was not studied. We found that the thermal denaturation of CXCR4 is a second-order reaction. This was unexpected, as the heat inactivation of several other G protein-coupled receptors (rhodopsin [80]–[82], phoborhodopsin [83], 5-HT_4_ receptor [84] and M_1_ muscarinic acetylcholine receptor [66]) proceeds through first-order reactions.

Various models have been proposed to explain second-order thermal denaturation reactions of proteins [84]. Two models, the model of parallel reactions [85] and the series-type model [86]–[88], are usually applied. According to the model of parallel reactions, two (or more) protein isoforms (E_1_, E_2_, …), each with a different level of heat resistance, exist. These isoforms are converted to inactive forms (I_n_) in parallel first-order reactions at rates governed by the constants k_n_:
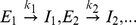
In the simplest case involving only two isoforms, the fraction (F) of the protein in the native conformation at time t is given as follows:
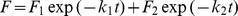
where F_1_ and F_2_ are the fractions of the native protein in each isoform at time t = 0. Note that, if F is normalized (F ≡ 1 at t = 0), then F_1_+F_2_ = 1.

According to a series-type model, protein inactivation proceeds in the following manner:
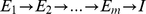
where E_2_, …, E_m_ are intermediate protein conformations, I is the inactivated conformation, and the first-order reactions E_1_→E_2_, …, E_m_→I proceed at rates described by the rate constants k_1_, …, k_m_. In the simplest case involving two steps (E_1_→E_2_→I) where the protein proceeds to complete inactivation in the final conformation,
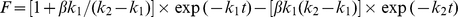
where β is the time-independent constant [88].

The equations for both the two-isoform parallel reaction model and the two-step series-type model can be written [67]:
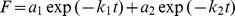
where a_1_ and a_2_ are constants. To estimate k_1_ and k_2_, we fitted the experimental data on the normalized 12G5 reactivity with CD4/CXCR4-proteoliposomes at various times after 60°C incubation to this equation. (The 1-min incubation time point at 60°C was excluded because it is too short compared with the time required for the sample to reach 60°C). This yielded the following results: k_1_ = 0.046 min^−1^ and k_2_ = 0.376 min^−1^ (r^2^ = 0.9993), which correspond to respective half-lives of 15.0 and 0.26 minutes for each of the two reactions.

Even in cell lysates, most or all of the CXCR4 molecules are dimeric [89]. Dimerization of CXCR4 provides a natural explanation of the observed second-order kinetics of denaturation. Dimeric CXCR4, by virtue of a greater number of molecular contacts with the partner subunit, is expected to exhibit better stability, as has been observed for other protein dimers ([90]–[95] and references therein). Thus, the series-type model, in which most or all of the CXCR4 starts out as the more heat-stable dimer, but upon incubation at 60°C, converts into more labile monomers, explains the available data. Indeed, dissociation [96], [97] or formation [98]–[100] of protein oligomers has been proposed as a possible mechanism underlying second-order kinetics of protein denaturation and renaturation, respectively. Although weaker than CXCR4 dimerization, the previously described CXCR4-CD4 interactions [101] could also contribute to the observed kinetics of CXCR4 denaturation.

We also evaluated the thermal denaturation of CD4 in the CD4/CXCR4-proteoliposomes. The heat-induced denaturation of other immunoglobulin superfamily members has been previously studied. CD4 in the CD4/CXCR4-proteoliposomes (activation energy of the denaturation reaction, E_a_ = 278 kJ/mol) was less thermostable than human and bovine immunoglobulin G (E_a_ = 315–393 kJ/mol) [102]–[106], bovine milk IgA (E_a_ = 567 kJ/mol), bovine milk IgM (E_a_ = 421 kJ/mol) [103], or the MHC class II molecule I-E^K^ (E_a_ = 427 kJ/mol) [107]. The multi-chain nature of immunoglobulin molecules may confer additional stability through inter-chain contacts [106] that would not be available to the single-chain CD4 molecule. It is also possible that the epitope for the Q4120 antibody in domain D1 of CD4, the integrity of which was used to monitor the native state of CD4 in the CD4/CXCR4-proteoliposomes, is more sensitive to heat than the immunoglobulin fold per se.

The order of the thermal denaturation of CD4 was found to be close to 1.3. Various reaction orders between 1 and 2 inclusive have been reported in the literature for the thermal denaturation of immunoglobulins [102]–[104], [106], [108], [109]. This is not surprising, given the multi-chain and oligomeric nature of the immunoglobulins. Indeed, it has been pointed out that the denaturation of proteins belonging to the immunoglobulin superfamily can be complex, involving several reactions with different temperature dependencies [110], [111]. Likewise, the weak dimerization of CD4 [6] and/or irreversible aggregation may contribute to the fractional (n = 1.3) order of the thermal denaturation of CD4.

Studies of the thermal sensitivity of membrane proteins like CD4 and CXCR4 in the context of different proteoliposome configurations should increase our understanding of how interactions with lipids and other proteins contribute to the structure and function of these biologically important molecules. Furthermore, proteoliposomes provide a useful system to assess mutagenic approaches designed to stabilize membrane proteins for structural studies [112]–[117].

## Materials and Methods

### Materials

1,2-dioleoyl-*sn*-glycero-3-phosphatidylcholine (DOPC), cholesterol, 1,2-dioleoyl-*sn*-glycero-3-phosphatidylethanolamine (DOPE), sphingomyelin (from porcine brain), and 1,2-dioleoyl-*sn*-glycero-3-phosphatidylethanolamine-N-(lissamine rhodamine B sulfonyl) (RhDOPE) were purchased from Avanti Polar Lipids, Inc. (Alabaster, AL). Ammonium sulfate (NH_4_)_2_SO_4_, sodium chloride (NaCl) and glycerol were purchased from Fisher Scientific (Pittsburgh, PA). UltraPure Tris, zeocin, Dulbecco's modified Eagle medium (DMEM), and fetal bovine serum (FBS) were purchased from Invitrogen (Carlsbad, CA). Protein A-Sepharose CL-4B beads were purchased from GE Healthcare (Uppsala, Sweden). Hygromycin B in PBS, polyethylene glycol (PEG) 1500 (50% wt/vol solution in 75 mM HEPES) and complete mini EDTA-free protease inhibitor cocktail tablets were purchased from Roche (Mannheim, Germany). Glycine was purchased from ICN Biomedicals (Irvine, CA). Dulbecco's phosphate-buffered saline (D-PBS) without Ca^+2^ or Mg^+2^, penicillin-streptomycin solution, trypsin-EDTA solution and G418 sulfate were purchased from Mediatech, Inc. (Herndon, VA). Sodium butyrate, EDTA, EGTA, and R-phycoerythrin (PE)-labeled anti-human CD4, clone Q4120 were purchased from Sigma-Aldrich (St. Louis, MO). CHAPSO [3-[(3-Cholamidopropyl) dimethylammonio]-2-hydroxy-1-propanesulfonate] was purchased from Anatrace, Inc. (Maumee, OH). PE-labeled anti-human CCR5, clone 2D7; PE-labeled anti-human CXCR4, clone 12G5; and PE-labeled anti-human CD4, clone RPA-T4 were purchased from BD Biosciences Pharmingen (San Jose, CA). PE-labeled anti-human CXCR4, clone 44717.111 was purchased from R&D Systems (Minneapolis, MN). CXCL12α (also known as SDF-1α) was purchased from PeproTech, Inc. (Rocky Hill, NJ). C12 peptide (VSKTETSQVAPA) was purchased from American Peptide Company (Sunnyvale, CA). HEPES was purchased from Boston BioProducts, Inc. (Worcester, MA). Human anti-gp120 monoclonal antibody 2G12 and small-molecule CXCR4 antagonist AMD3100 were obtained from the NIH AIDS Research and Reference Reagent Program (Germantown, MD). Soluble CD4 was produced in 293T cells after stable transfection or, in some cases, was purchased from ImmunoDiagnostics, Inc. (Woburn, MA). The 1D4 murine monoclonal antibody directed against the C9 peptide (TETSQVAPA) was obtained from the National Cell Culture Center (Minneapolis, MN). TAK779 was generously provided by Takeda Pharmaceuticals (Deerfield, IL). Compound A was generously provided by Merck (Whitehouse Station, NJ).

### Aqueous buffers

Solubilization buffer S1 had the following composition: 100 mM (NH_4_)_2_SO_4_, 20 mM Tris (pH 7.5), 10 vol % glycerol, 1% (weight∶volume) CHAPSO, and protease inhibitor cocktail (1 mini tablet per 10 ml). Solubilization buffer S2 had the following composition: 100 mM (NH_4_)_2_SO_4_, 20 mM Tris (pH 7.5), 1% (weight∶volume) CHAPSO, and protease inhibitor cocktail (1 mini tablet per 10 ml). Glycerol-free dialysis buffer had the following composition: 100 mM (NH_4_)_2_SO_4_ and 20 mM Tris (pH 7.5). Glycerol-containing dialysis buffer had the following composition: 100 mM (NH_4_)_2_SO_4_, 20 mM Tris (pH 7.5) and 10 vol % glycerol.

### Preparation of lipid mixture

Lipids (in chloroform solutions) were pooled in cryovials (a total of 13.5 µmole in each cryovial) and dried under vacuum until all of the solvent was removed. The lipid mixture from each cryovial was then resuspended in 1 ml D-PBS and sonicated using the Branson Sonifier 450 (Misonix, Inc., Farmingdale, NY). Sonicated lipid mixture was stored under argon at −30°C. Except as indicated below, the lipid mixture had (if not stated otherwise) the following composition: 35% DOPC, 30% DOPE, 15% sphingomyelin, and 20% cholesterol (so-called “nature's own” fusogenic lipid composition) [118]. The composition of the lipid mixture is given as the molar ratio between component lipids.

Preparations of CXCR4-proteoliposomes used in the dynamic light scattering experiments, in electron microscopy, and in the studies of antibody and AMD3100 binding to extruded proteoliposomes were made with the following lipid composition: 40 mol % DOPC, 40 mol % sphingomyelin, and 20 mol % cholesterol.

### 293T cells expressing CD4 containing the C9 tag

293T cells stably expressing human CD4 containing the C9 peptide (TETSQVAPA) at the C terminus were made by Christoph Grundner, using the pcDNA3.1+ plasmid (Invitrogen, Carlsbad, CA).

### Cf2Th cells expressing CXCR4 containing the C9 tag

Cf2Th cells stably expressing human CXCR4 containing the C9 peptide (TETSQVAPA) at the C terminus are described elsewhere [33].

### Cell culture

Cells were grown at 37°C with 5% CO_2_. CXCR4-free Cf2Th cells and CD4-free 293T cells were grown in DMEM supplemented with 10% fetal bovine serum (FBS), 100 IU/ml penicillin and 100 µg/ml streptomycin (complete DMEM). Cf2Th cells stably expressing human CXCR4 and CD4 were grown in complete DMEM containing 0.2 mg/ml zeocin and 0.2 mg/ml hygromycin B. Cf2Th cells stably expressing human CXCR4 containing C9 peptide at the C terminus and 293T cells stably expressing human CD4 containing C9 peptide at the C terminus were grown in complete DMEM containing 0.5 mg/ml G418.

### Preparation of cell lysates

Cell lysates were prepared the following way. Cells grown to full confluency in a 150-mm dish were treated for 24 hours with complete DMEM containing 3 mM sodium butyrate prior to harvest, detached by treatment with D-PBS/5 mM EDTA, pelleted, washed in D-PBS, again pelleted, and stored at −30°C until needed. Frozen cells were solubilized with S1 solubilization buffer (∼5×10^7^ cells in 1 ml of buffer) at 4°C for 45 minutes on a rocking platform. Cell debris were pelleted by centrifugation for 30 minutes at 16,000×g at 4°C, and the cleared lysate was stored at −30°C. For the preparation of the CXCR4-containing cell lysate, Cf2Th cells stably expressing human CXCR4 containing the C9 peptide at the C terminus were used. For the preparation of the CD4-containing cell lysate, 293T cells stably expressing human CD4 containing the C9 peptide at the C-terminus were used. For the preparation of CXCR4-free cell lysate, Cf2Th cells were used. For the preparation of CD4-free cell lysate, 293T cells were used.

### Formation of proteoliposomes

Approximately 1.5 g of Protein A-Sepharose CL-4B beads were washed 4 times with 45 ml of water and then resuspended in ∼7 ml of D-PBS/0.01% sodium azide and stored at 4°C.

One volume of the solution of Protein A-Sepharose CL-4B beads was washed twice with solubilization buffer S1 and then resuspended in 0.4 volumes of the solution of the 1D4 antibody at a concentration of 1.1 mg/ml (a mixture of 0.08 volumes of the stock solution (5.5 mg/ml) of 1D4 and 0.32 volumes of solubilization buffer S1). Then beads were incubated overnight at 4°C on a rocking platform. The beads were then washed three times with solubilization buffer S1, and resuspended in 0.5 volumes of the same buffer. This bead suspension was aliquoted, 400 µl in each aliquot. To each aliquot, 1 ml of cell lysate was added. (For the preparation of CXCR4-proteoliposomes or CD4-proteoliposomes, CXCR4-containing lysate or CD4-containing lysate, respectively, was used. For the preparation of CD4/CXCR4-proteoliposomes, a mixture of 33.3 vol % of CXCR4-containing lysate and 66.7 vol % of CD4-containing lysate was used). The beads were incubated with the cell lysate for at least 2 hours at 4°C on a rocking platform.

Next, the beads were washed 5 times with solubilization buffer S2 and then CXCR4 (and/or CD4) was eluted in the following manner. Beads in each aliquot were incubated for 1 hour at room temperature with 200 µl of a 200 µg/ml solution of peptide C12 (a mixture of 160 µl of the solubilization buffer S2 and 40 µl of the stock aqueous solution (1 mg/ml) of C12 peptide) on a rocking platform. The beads were pelleted, supernatants containing the eluted material were collected, and a second round of elution was carried out. The two eluted solutions were combined. After centrifugation to remove the beads, supernatants were collected.

A suspension consisting of the eluted material and the lipid mixture in D-PBS was prepared (90 vol % of eluted material and 10 vol % lipid mixture in D-PBS). This suspension was dialyzed overnight at 4°C against glycerol-free dialysis buffer (unless stated otherwise), using a 10-kDa molecular weight cutoff (Slide-A-Lyzer 10 K; Pierce, Rockford, IL) to remove detergent and the C12 peptide and allow the formation of proteoliposomes. After five freeze-thaw cycles, proteoliposomes were stored at 4°C.

The CXCR4-proteoliposomes used in the dynamic light scattering experiments, in electron microscopy, and in the studies of antibody and AMD3100 binding to extruded proteoliposomes were prepared using solubilization buffer S1 instead of S2 and dialysis against glycerol-containing buffer.

Protein-free liposomes were prepared using dialysis of the mixture of 90 vol % solubilization buffer S1 and 10 vol % lipid mixture in D-PBS against glycerol-containing dialysis buffer.

### Formation of extruded proteoliposomes

Proteoliposomes (prepared as described above) were extruded several times through two polycarbonate Nucleopore filters of 100-nm pore size in a pressure extruder (Northern Lipids, Burnaby, BC, Canada) to produce large unilamellar vesicles. All preparations were extruded at 37°C; the temperature during extrusion was maintained with a circulating water bath (Voigt Global Distribution, Lawrence, KS). Liposomes prepared by extrusion through 100-nm pore size filters were homogeneous and fusogenic ([119], and references therein).

### Characterization of proteoliposome size

The size of non-extruded and extruded CXCR4-proteoliposomes was determined by dynamic light scattering (using a Coulter N4 Plus submicron particle analyzer) and electron microscopy.

Electron microscopy was carried out as follows. Five µl of each sample was absorbed to a formvar/carbon-coated grid for 1 minute and excess liquid was blotted off using Whatman #1 filter paper. The grid was stained with 1% uranyl acetate for 1 minute, blotted with a filter paper again and examined in a Tecnai Spirit BioTwin transmission electron microscope at the Electron Microscopy Core facility (Harvard Medical School, Boston, MA).

### Analysis of protein composition of the proteoliposomes

The protein composition of proteoliposomes was analyzed by silver staining on an SDS-polyacrylamide gel as follows. Proteoliposomes suspensions were incubated at 37°C for 30 minutes in 1× SDS loading buffer containing 5% β-mercaptoethanol (β-ME) from Bio-Rad Laboratories, Inc. (Hercules, CA) (reducing conditions) or in loading buffer without β-ME (non-reducing conditions). Samples were run on SDS-polyacrylamide gels and analyzed by silver staining using GelCode SilverSNAP (Pierce, Rockford, IL) kit, as described by the manufacturer.

### Antibody binding to CD4/CXCR4-proteoliposomes

The binding of anti-CXCR4 and anti-CD4 antibodies to CD4/CXCR4-proteoliposomes was analyzed by fluorescence-activated cell sorting (FACS). Proteoliposomes were incubated for 45 minutes at room temperature in D-PBS/3% fetal bovine serum (FBS) containing varying concentrations of R-phycoerythrin (PE)-labeled monoclonal antibody, in a volume of 100 µl. After this incubation, 200 µl of D-PBS/3% FBS was added to each sample. All samples were analyzed with a FACScan flow cytometry using CellQuest software (Becton Dickinson, San Jose, CA) in the FL2 channel. Fluorescence of proteoliposomes incubated with varying concentrations of PE-labeled anti-CCR5 monoclonal antibody 2D7 was subtracted, as a baseline, from all fluorescence measurements obtained with specific antibodies.

### CXCL12 binding to CD4/CXCR4-proteoliposomes and Cf2Th-CD4/CXCR4 cells

The binding of the CXCR4 ligand CXCL12 to CD4/CXCR4-proteoliposomes was analyzed by FACS using a competition assay [46], [55]. Proteoliposomes were incubated in D-PBS/3% FBS containing various concentrations of CXCL12 in a final volume of 100 µl. Incubation for 20 minutes at room temperature in the absence of antibody, was followed by incubation for 45 minutes at room temperature, in the presence of 0.25 µg/ml of PE-labeled anti-CXCR4 monoclonal antibody 12G5. After this incubation, 200 µl of D-PBS/3% FBS was added to each sample. All samples were analyzed with a FACScan flow cytometry as described above. Fluorescence of proteoliposomes incubated with 0.125 µg/ml of PE-labeled anti-CCR5 monoclonal antibody 2D7, in the absence of CXCL12, was subtracted, as a baseline, from all fluorescence measurements obtained with the 12G5 antibody.

The ability of CXCL12 to compete for the binding of the 12G5 antibody to Cf2Th-CD4/CXCR4 cells was studied as described above for proteoliposomes.

In control experiments, the binding of 0.12 µg/ml of PE-labeled anti-CD4 monoclonal antibody Q4120, 0.125 µg/ml of PE-labeled anti-CD4 monoclonal antibody RPA-T4, and 0.25 µg/ml of PE-labeled anti-CXCR4 monoclonal antibody 44717.111 to CD4/CXCR4-proteoliposomes was studied in the absence of CXCL12 and in the presence of 500 nM of CXCL12.

### AMD3100 binding to CD4/CXCR4-proteoliposomes and Cf2Th-CD4/CXCR4 cells

The binding of the small-molecule CXCR4 antagonist AMD3100 to CD4/CXCR4-proteoliposomes was analyzed by FACS using a competition assay [47], [55]. Proteoliposomes were incubated in D-PBS/3% FBS containing various concentrations of AMD3100 in a final volume of 100 µl. Incubation for 20 minutes at room temperature, in the absence of antibody, was followed by incubation for 45 minutes at room temperature in the presence of 1.25 µg/ml of PE-labeled anti-CXCR4 monoclonal antibody 12G5. After this incubation, 200 µl of D-PBS/3% FBS was added to each sample. All samples were analyzed with a FACScan flow cytometry as described above. Fluorescence of proteoliposomes incubated with 0.625 µg/ml of PE-labeled anti-CCR5 monoclonal antibody 2D7, in the absence of AMD3100, was subtracted, as a baseline, from all fluorescence measurements obtained with the 12G5 antibody.

The ability of AMD3100 to compete for the binding of the 12G5 antibody to Cf2Th-CD4/CXCR4 cells was studied as described above for proteoliposomes.

In control experiments, the binding of 0.6 µg/ml of PE-labeled anti-CD4 monoclonal antibody Q4120 and 0.625 µg/ml of PE-labeled anti-CD4 monoclonal antibody RPA-T4 to CD4/CXCR4-proteoliposomes and Cf2Th-CD4/CXCR4 cells was studied in the absence of AMD3100 and in the presence of 0.241 µM of AMD3100.

In other control experiments, the binding of 1.25 µg/ml of PE-labeled anti-CXCR4 monoclonal antibody 12G5 to CD4/CXCR4-proteoliposomes and Cf2Th-CD4/CXCR4 cells was studied in the absence of any ligand and in the presence of 2.4 µM TAK779 or Compound A, small-molecule CCR5 antagonists.

### Antibody and AMD3100 binding to extruded CXCR4-proteoliposomes

The binding of anti-CXCR4 monoclonal antibodies 12G5 and 44717.111 and the small-molecule CXCR4 antagonist AMD3100 to extruded CXCR4-proteoliposomes (and, as a control, to non-extruded CXCR4-proteoliposomes and to extruded and non-extruded protein-free liposomes) was analyzed in the following manner.

Liposomes were incubated with 25 µl of 4-µm-diameter aldehyde/sulfate latex beads (Interfacial Dynamics, Portland, OR) in a final volume of 150 µl for 55 minutes at room temperature, followed by a 90-minute incubation in 1 ml D-PBS with gentle shaking. The reaction was stopped by incubation for 30 minutes in D-PBS supplemented with 100 mM glycine. Liposome-coated beads were washed three times with D-PBS/3% FBS and resuspended in 50 µl of D-PBS/3% FBS.

For the study of the antibody binding to liposomes, the liposome-coated beads were incubated for 45 minutes at room temperature in D-PBS/3% FBS containing 0.625 µg/ml of PE-labeled anti-CXCR4 monoclonal antibody 12G5 or 1.25 µg/ml of PE-labeled anti-CXCR4 monoclonal antibody 44717.111, in a volume of 100 µl. For the study of AMD3100 binding to liposomes using the competition assay, the liposome-coated beads were incubated in D-PBS/3% FBS containing 0.241 µM AMD3100 in a final volume of 100 µl. Incubation for 20 minutes at room temperature, in the absence of antibody, was followed by incubation for 45 minutes at room temperature in the presence of 0.625 µg/ml of PE-labeled anti-CXCR4 monoclonal antibody 12G5.

After incubation, 500 µl of D-PBS/3% FBS was added to each sample. All samples were analyzed with a FACScan flow cytometry as described above. Fluorescence of liposome-coated beads incubated with 0.625 µg/ml of PE-labeled anti-CCR5 monoclonal antibody 2D7, in the absence of AMD3100, was subtracted, as a baseline, from all fluorescence measurements obtained with CXCR4-specific antibodies.

### Production of gp120 glycoprotein

Soluble HIV-1_HXBc2_ gp120 glycoprotein was prepared in the following manner. Approximately 3.5×10^6^ 293T cells were seeded in a T75 tissue culture flask one day before transfection. Cells were co-transfected with 9 µg of pSVIIIenv and 1 µg of pLTR-Tat plasmids using the Polyfect transfection reagent (Qiagen, Germantown, MD). The supernatant was harvested 48 hours later, cleared by centrifugation at 2,000 rpm for 5 minutes, and stored at 4°C. The amount of gp120 was quantified by Western blot analysis.

### Binding of soluble gp120 to CXCR4-proteoliposomes and CD4-proteoliposomes

The binding of soluble HIV-1_HXBc2_ gp120 to CXCR4-proteoliposomes and CD4-proteoliposomes was analyzed by FACS. Proteoliposomes were incubated for 2 hours at 37°C in a total volume of 100 µl, containing HIV-1_HXBc2_ gp120. As a control, proteoliposomes were incubated for 2 hours at 37°C without gp120. Incubation of CXCR4-proteoliposomes was carried out in the presence of 80 µg/ml of soluble CD4 (or, as a control, in the absence of soluble CD4). Proteoliposomes were then centrifuged for 10 minutes at 20,800×g and resuspended in 100 µl of D-PBS/3% FBS containing 5 µg/ml of the human monoclonal antibody 2G12, which recognizes a carbohydrate epitope on the gp120 outer domain [120], [121]. Proteoliposomes were incubated for 45 minutes at room temperature, centrifuged for 10 minutes at 20,800×g and resuspended in 100 µl of D-PBS/3% FBS containing 5 µl of goat anti-human PE-labeled IgG antibody (Jackson ImmunoResearch Laboratories, West Grove, PA). Proteoliposomes were then incubated for 45 minutes at room temperature. After this incubation, 200 µl of D-PBS/3% FBS was added to each sample. All samples were analyzed with a FACScan flow cytometry as described above.

### PEG-mediated lipid mixing between cells and CXCR4-proteoliposomes

CXCR4-proteoliposomes (lipid composition: 35 mol % DOPC, 25 mol % DOPE, 15 mol % sphingomyelin, 20 mol % cholesterol, 5 mol % RhDOPE), prepared using solubilization buffer S1 instead of S2 and glycerol-containing dialysis buffer, were used. Polyethylene glycol (PEG)-mediated lipid mixing between 293T cells and CXCR4-proteoliposomes was studied in the following manner. Cells were detached by treatment with D-PBS/5 mM EDTA, pelleted, washed in D-PBS, again pelleted, and resuspended in D-PBS/3% FBS. Samples (total volume 360 µl each) containing D-PBS, 100 µl of cell suspension, 10 µl of the suspension of CXCR4-proteoliposomes and 120 µl of the PEG solution in HEPES buffer (or, as a control, not containing PEG) were incubated for 1 hour at 37°C. After this incubation, samples were analyzed with a FACScan flow cytometry as described above.

### Plasmids and transfection

The plasmids that express the HIV-1_KB9_ envelope glycoprotein [122] and the HIV-1 Tat protein, and a plasmid containing the deleted ΔKS env gene were prepared using the Plasmid Maxi Kit (Qiagen, Germantown, MD).

To prepare cells expressing the HIV-1_KB9_ envelope glycoproteins, 293T cells were co-transfected in T75 tissue culture flasks with the HIV-1_KB9_ Env-expressing plasmid and an HIV-1 Tat-expressing plasmid using the calcium phosphate precipitation method (Invitrogen, Carlsbad, CA). Control cells were prepared in the same manner, except that the plasmid containing the deleted ΔKS env gene was used instead of the Env-expressing plasmid.

Surface expression of the HIV-1 envelope glycoproteins on Env-transfected 293T cells was analyzed by FACS in the following manner. Cells were detached by treatment with D-PBS/5 mM EDTA, pelleted, washed in D-PBS, pelleted again, and resuspended in 50 µl of D-PBS/3% FBS containing 5 µg/ml of human monoclonal antibody 2G12, which recognizes a carbohydrate epitope on the gp120 outer domain [120], [121]. Cells were incubated for 1 hour at room temperature, pelleted and washed in D-PBS/3% FBS twice, and then pelleted and resuspended in 100 µl of D-PBS/3% FBS containing 2 µl of goat anti-human PE-labeled IgG antibody (Jackson ImmunoResearch Laboratories, West Grove, PA). Cells were then incubated for 1 hour at room temperature. After this incubation, cells were pelleted, washed in D-PBS/3% FBS, pelleted, resuspended in 500 µl of D-PBS/3% FBS and then analyzed with a FACScan flow cytometry as described above.

### Cell-proteoliposome interactions

For the study of cell-proteoliposome interactions, transfected 293T cells and fluorescently labeled extruded proteoliposomes were used. Interaction of cells transfected with plasmids that express the functional HIV-1_KB9_ envelope glycoproteins or that contains the deleted ΔKS env gene with either CD4/CXCR4-proteoliposomes or CD4-proteoliposomes was studied.

Extruded CD4/CXCR4-proteoliposomes and CD4-proteoliposomes were prepared using a lipid mixture of the following composition: 35 mol % DOPC, 25 mol % DOPE, 15 mol % sphingomyelin, 20 mol % cholesterol, and 5 mol % RhDOPE. Hence, these proteoliposomes contain a fluorescent rhodamine-labeled lipid probe in a self-quenching concentration.

Transfected 293T cells were harvested 48 hours after transfection: cells were detached by treatment with D-PBS/5 mM EDTA, pelleted, washed in D-PBS, pelleted again, and resuspended in complete DMEM. The concentration of cells was adjusted to 3×10^5^ cells/ml, and 3×10^5^ cells were seeded in each well of a 24-well plate. Cells were incubated at 37°C with 5% CO_2_ overnight.

The following day, 80 µl of proteoliposome suspension (or, as a control, D-PBS) was added to each well of cells. Proteoliposomes were incubated with cells for 8.5 hours at 37°C with 5% CO_2_. The medium was then removed, and cells were harvested in 1 ml D-PBS, pelleted and washed in D-PBS twice, and then pelleted and resuspended in 500 µl of D-PBS. All samples were then analyzed with a FACScan flow cytometry as described above. Each of the experimental conditions was studied in parallel triplicate experiments and the results averaged.

### Thermal denaturation of CXCR4 and CD4 molecules incorporated in CD4/CXCR4-proteoliposomes

After the incubation of CD4/CXCR4-proteoliposomes at different temperatures for various periods of time, the binding of anti-CXCR4 and anti-CD4 antibodies to these proteoliposomes was analyzed by FACS. Suspensions of proteoliposomes were incubated at different temperatures for different periods of time, and immediately placed on ice. Afterwards, proteoliposomes were incubated for 45 minutes at room temperature in D-PBS/3% FBS containing 1.25 µg/ml of the PE-labeled anti-CXCR4 monoclonal antibody 12G5, 0.6 µg/ml of the PE-labeled anti-CD4 monoclonal antibody Q4120, or 0.625 µg/ml of the PE-labeled anti-CCR5 monoclonal antibody 2D7 in a volume of 100 µl. After this incubation, 200 µl of D-PBS/3% FBS was added to each sample. All samples were analyzed with a FACScan flow cytometry as described above. The fluorescence of proteoliposomes incubated with the 2D7 antibody was subtracted, as a baseline, from all fluorescence measurements obtained with the 12G5 and Q4120 antibodies. Each experiment was performed in triplicate and employed proteoliposomes from three separate preparations.

### Kinetic analysis of the protein denaturation reaction

The rate of the protein denaturation reaction at particular temperature can be generally described by the formula:
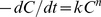
(1)where C is the concentration of undenatured protein at time t, n is the reaction order, and k is the reaction rate constant at this temperature [123]. If n = 1 (i. e., if denaturation follows first-order kinetics), integration of (1) yields:
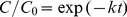
(2)where C_0_ is the value of C at time t = 0 (i. e., the initial protein concentration).

If n≠1, integration of (1) yields:
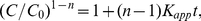
(3)where K_app_ = kC_0_^n−1^; here, K_app_ is the apparent denaturation rate constant, with k being the true rate constant.

In our experiments, the initial concentration of protein C_0_ is unknown and, hence, if the denaturation reaction does not follow first-order kinetics, we cannot find the true denaturation rate constant k. However, we assume that C/C_0_ is equal to F, which is defined as the specific fluorescence of proteoliposomes associated with the binding of a conformation-dependent antibody, normalized to the specific fluorescence observed for proteoliposomes that were not heated. Therefore, equation (2) can be rewritten as:
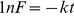
(4)


Thus, if thermal inactivation is a first-order reaction, the value of k can be obtained from the plot of ln F versus t.

Likewise, equation (3) can be rewritten as
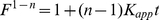
(5)


The function F^1−n^ versus t should be a straight line (i. e., the correlation coefficient r^2^ should be close to 1), and the ordinate intercept b (at t = 0) of this line should be close to 1, if the process follows the estimated reaction order. Then, if (F^1−n^−1)/(n−1) is plotted versus t, the value of K_app_ equals the slope of the plotted line.

Half-lives and decimal reduction times of the thermal denaturation can be calculated for each temperature. The half-lives (t_1/2_) of the thermal denaturation (time required for 50% denaturation at constant temperature) can be calculated using the formula:
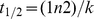
(6)for first-order kinetics, and
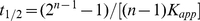
(7)for non-first-order kinetics.

Similarly, D values (decimal reduction time, i. e., time required for 90% denaturation at constant temperature [102], [103]) can be calculated using the formula
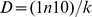
(8)for first-order kinetics, and
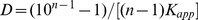
(9)for non-first-order kinetics.

### Activation energies and Z values associated with thermal denaturation of proteins

Two models, the Arrhenius model and the Bigelow model, have been used to describe the thermal denaturation of proteins. The Arrhenius model is based on the assumption that the logarithm of the denaturation reaction rate constant k decreases linearly with an increase in reciprocal temperature. By contrast, in the Bigelow model, the logarithm of k increases linearly with an increase in temperature. By using the Arrhenius model, the activation energy E_a_ of the denaturation reaction can be calculated. By using the Bigelow model (also known as thermal death time (TDT) model), one can calculate the Z value, a measure of the temperature dependence of the denaturation reaction rate.

Strictly speaking, the assumptions underlying these two models contradict each other [124]. However, the general belief is that both methods can be used satisfactorily, within a relatively short temperature range [125]. Often, as in this study, both models are employed.

According to the Arrhenius model, the absolute temperature T and the denaturation constant k are related according to the empirical Arrhenius equation [123]

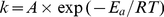
(10)where E_a_ is the activation energy of the denaturation reaction, A is the pre-exponential factor (sometimes called the frequency factor) which is assumed to be temperature-independent, and R = 8.3145 J/(K·mol) = 1.987 cal/(K·mol) is the universal gas constant.

Taking natural logarithms, equation (10) becomes:
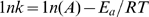
(11)


For non-first-order kinetics, equation (11) can be modified as:
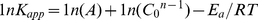
(12)


When the natural logarithm of the denaturation rate constant (or natural logarithm of the apparent rate constant) is plotted against reciprocal temperature 1/T according to formulae (11) and (12), an Arrhenius plot is obtained. For a single rate-limited thermally activated process, an Arrhenius plot gives a straight line, and the value of E_a_ can be obtained from the slope -E_a_/R by regression analysis.

According to the Bigelow model, by using D values, the Z value [102], [103], which is the temperature increase required for a 90% decrease in D value, can be calculated by regression analysis from the negative reciprocal slope of the line when the log_10_D values versus temperature are plotted.

The inactivation temperature (T_i_) can be defined as the temperature when the half-life t_1/2_ of thermal denaturation is equal to 10 minutes [80], [82], [126]. The value of T_i_ can be found by regression analysis of the line when the log_10_(t_1/2_) values versus temperature are plotted. This plot can be approximated as a straight line
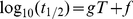
(13)and the value of T_i_ can be calculated as T_i_ = (1−f)/g.

All fittings and linear regressions were carried out using the Origin software package.

## Supporting Information

Figure S1 Size of proteoliposomes. The non-extruded (A) and extruded (B) CXCR4-proteoliposomes were analyzed by dynamic light scattering (left) and by electron microscopy (right). The dynamic light scattering analysis reveals the diameters of the proteoliposomes in the non-extruded and extruded preparations; the average diameter of the proteoliposomes in each population is noted beneath the figures. The magnification of the electron micrographs differ, as indicated by the scale bars.
(1.52 MB TIF)

Figure S2 Protein composition of the proteoliposomes. CXCR4-proteoliposomes (CXCR4-PL), CD4-proteoliposomes (CD4-PL), or control proteoliposomes (PL) were lysed and analyzed under nonreducing (−β-ME) or reducing (+β-ME) conditions by SDS-PAGE. The gel was silver stained.
(0.65 MB TIF)

Figure S3 Polyethylene glycol (PEG)-mediated fusion of cells and proteoliposomes. The mean fluorescence intensity of 293T cells mixed with rhodamine-labeled non-extruded or extruded CXCR4-proteoliposomes is shown after treatment with PEG or, as a control, in the absence of PEG.
(0.15 MB TIF)

Figure S4 Association of CD4-proteoliposomes and CD4/CXCR4-proteoliposomes with cells expressing HIV-1 envelope glycoproteins. The median fluorescence intensity of 293T cells expressing the HIV-1_KB9_ envelope glycoproteins (KB9) or transfected with a plasmid containing the deleted ΔKS env gene is shown, following incubation with D-PBS or rhodamine-labeled CD4-proteoliposomes or rhodamine-labeled CD4/CXCR4-proteoliposomes. The data shown represent the means and standard deviations derived from triplicate experiments.
(0.15 MB TIF)

Table S1 Inhibition of antibody binding to CD4/CXCR4-proteoliposomes by ligands.
(0.03 MB DOC)
