# Supplementary figures and images for: Thermal Stability of the Human Immunodeficiency Virus Type 1 (HIV-1) Receptors, CD4 and CXCR4, Reconstituted in Proteoliposomes
Source: PLoS One. 2010 Oct 13;5(10):e13249. doi: 10.1371/journal.pone.0013249 (PMC2954141; doi:10.1371/journal.pone.0013249)

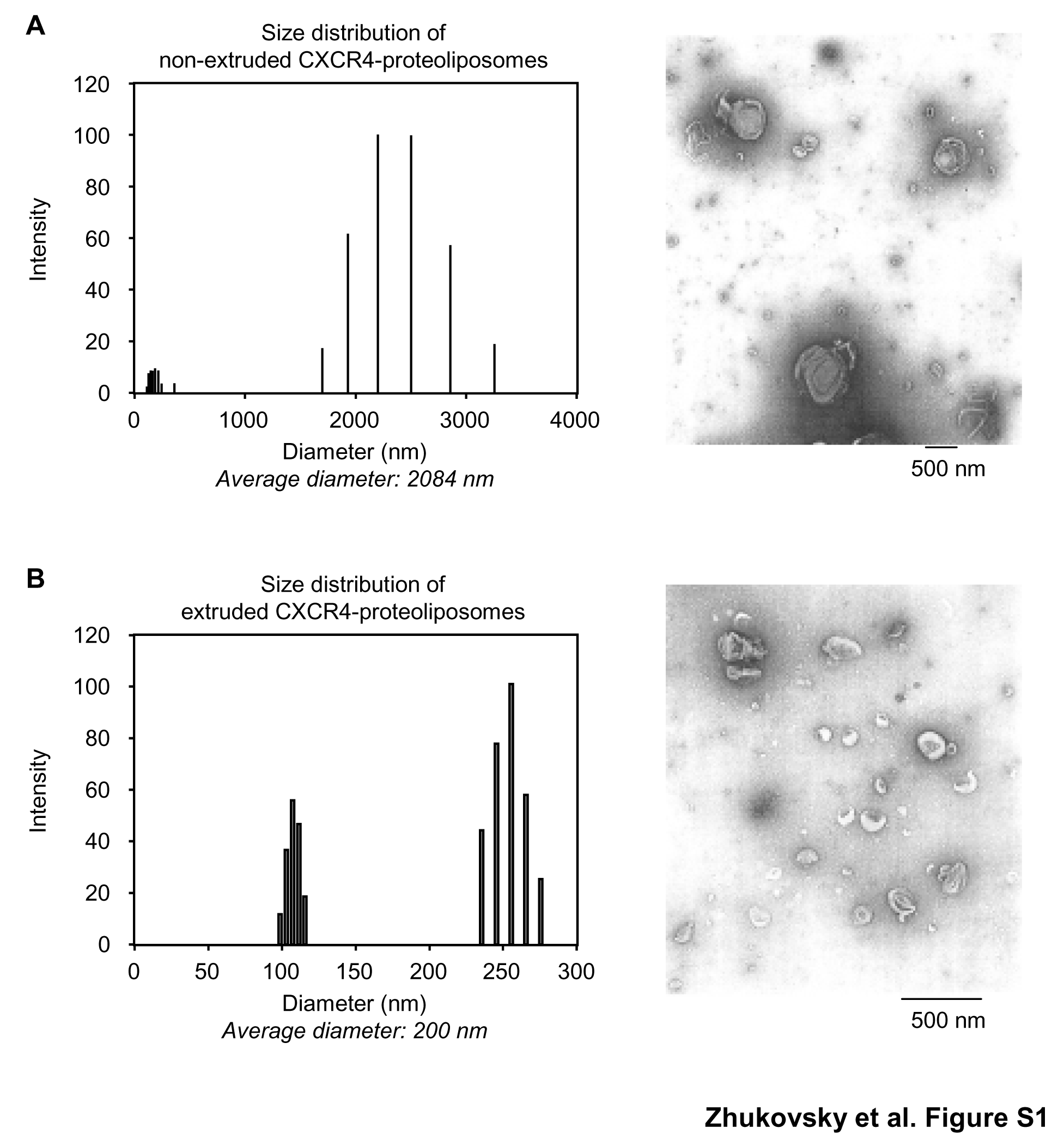

Supplement: Figure S1 — Size of proteoliposomes. The non-extruded (A) and extruded (B) CXCR4-proteoliposomes were analyzed by dynamic light scattering (left) and by electron microscopy (right). The dynamic light scattering analysis reveals the diameters of the proteoliposomes in the non-extruded and extruded preparations; the average diameter of the proteoliposomes in each population is noted beneath the figures. The magnification of the electron micrographs differ, as indicated by the scale bars. (1.52 MB TIF) [file pone.0013249.s001.tif]

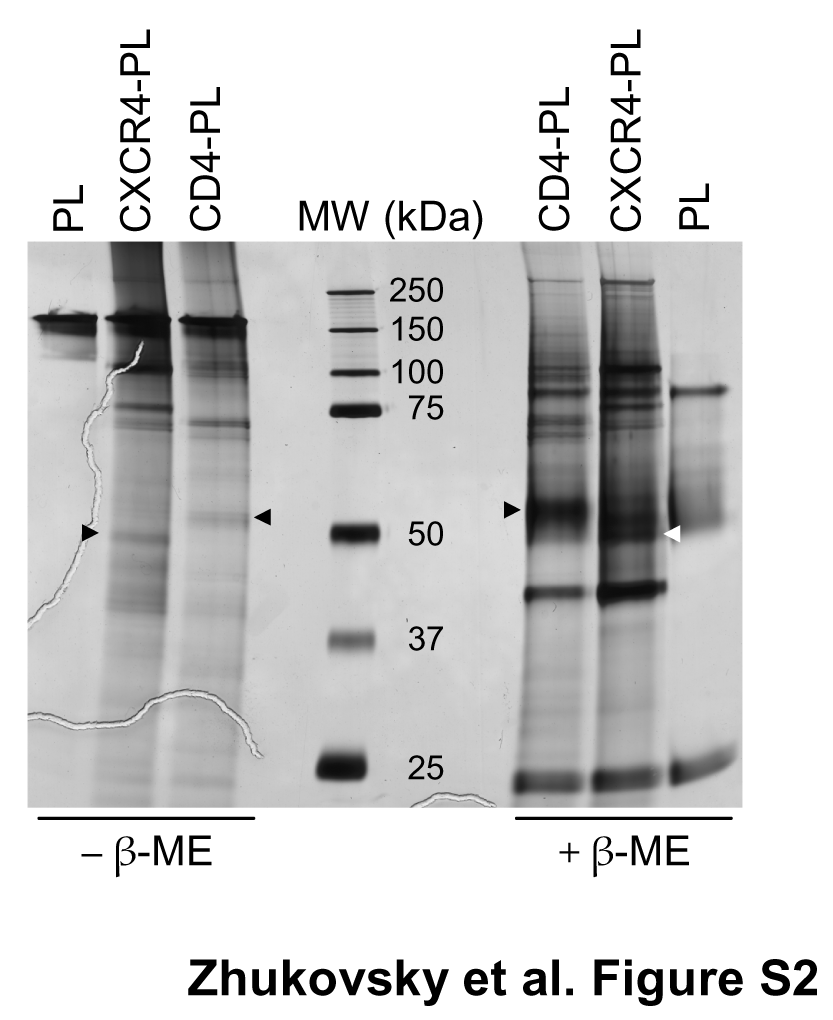

Supplement: Figure S2 — Protein composition of the proteoliposomes. CXCR4-proteoliposomes (CXCR4-PL), CD4-proteoliposomes (CD4-PL), or control proteoliposomes (PL) were lysed and analyzed under nonreducing (−β-ME) or reducing (+β-ME) conditions by SDS-PAGE. The gel was silver stained. (0.65 MB TIF) [file pone.0013249.s002.tif]

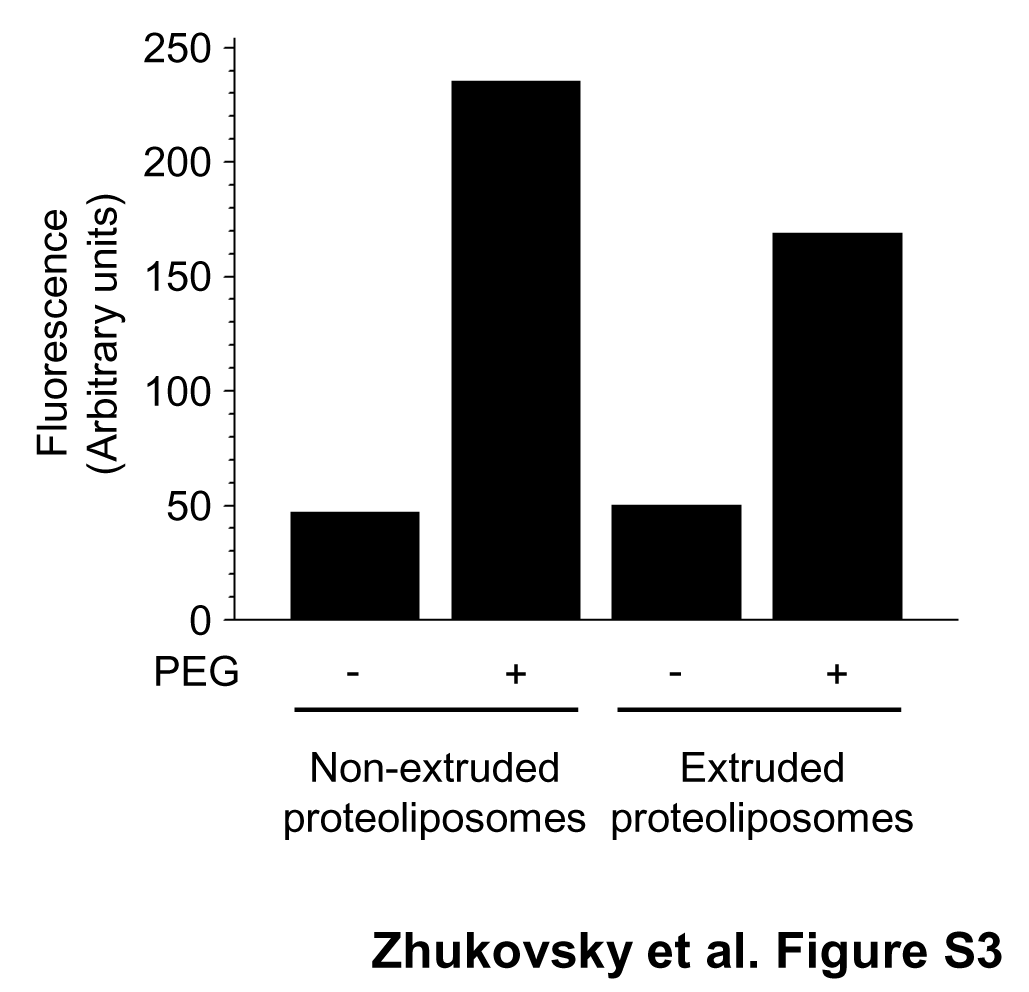

Supplement: Figure S3 — Polyethylene glycol (PEG)-mediated fusion of cells and proteoliposomes. The mean fluorescence intensity of 293T cells mixed with rhodamine-labeled non-extruded or extruded CXCR4-proteoliposomes is shown after treatment with PEG or, as a control, in the absence of PEG. (0.15 MB TIF) [file pone.0013249.s003.tif]

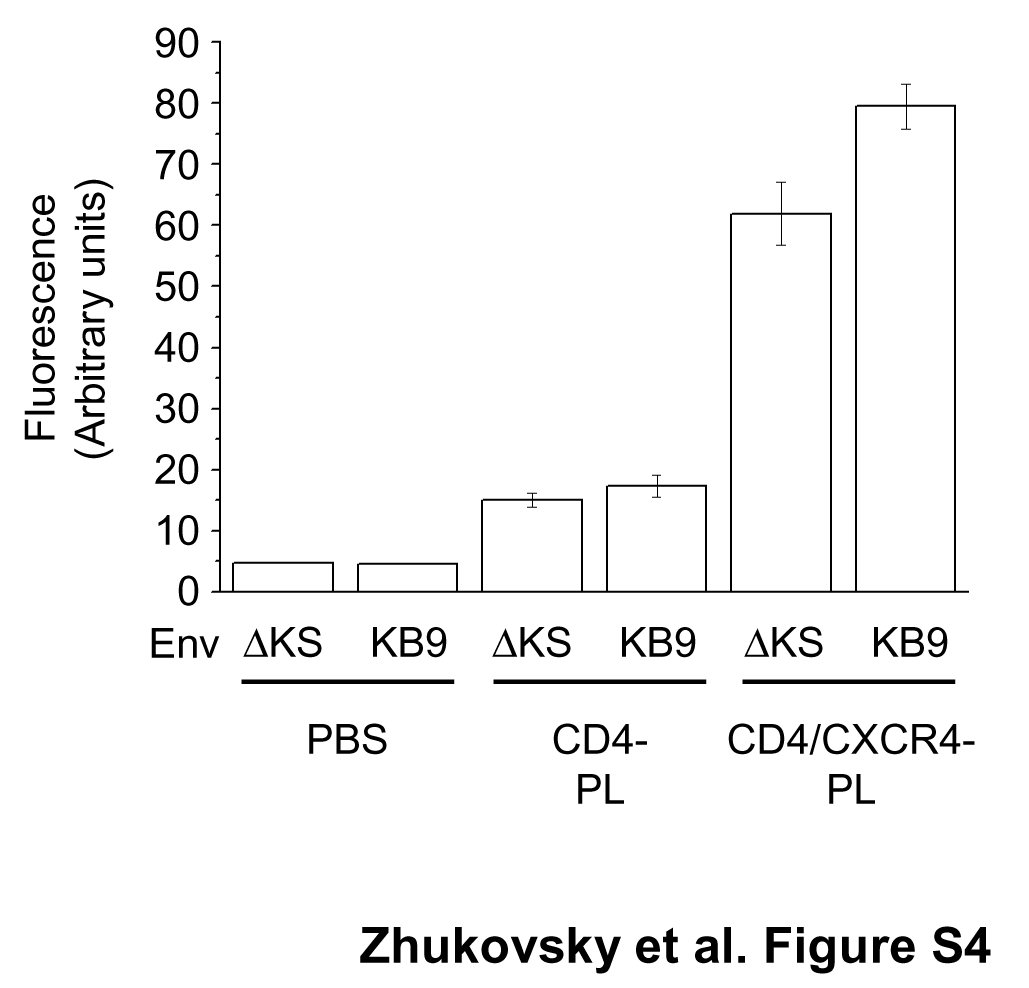

Supplement: Figure S4 — Association of CD4-proteoliposomes and CD4/CXCR4-proteoliposomes with cells expressing HIV-1 envelope glycoproteins. The median fluorescence intensity of 293T cells expressing the HIV-1KB9 envelope glycoproteins (KB9) or transfected with a plasmid containing the deleted ΔKS env gene is shown, following incubation with D-PBS or rhodamine-labeled CD4-proteoliposomes or rhodamine-labeled CD4/CXCR4-proteoliposomes. The data shown represent the means and standard deviations derived from triplicate experiments. (0.15 MB TIF) [file pone.0013249.s004.tif]
