# Supplementary material for: Thermal Stability of the Human Immunodeficiency Virus Type 1 (HIV-1) Receptors, CD4 and CXCR4, Reconstituted in Proteoliposomes
Source: PLoS One. 2010 Oct 13;5(10):e13249. doi: 10.1371/journal.pone.0013249 (PMC2954141; doi:10.1371/journal.pone.0013249)
Supplement: Table S1 — Inhibition of antibody binding to CD4/CXCR4-proteoliposomes by ligands. (0.03 MB DOC) [file pone.0013249.s005.doc]

**Table S1. Inhibition of antibody binding to CD4/CXCR4-proteoliposomes by ligands**

| **Labeled Antibody** | **Antibody Target** | **Competitor (Target)^a^** | **Mean Fluorescence Intensity^b^** |
| --- | --- | --- | --- |
| Q4120 | CD4 | None | 482 |
|  |  | AMD3100 (CXCR4) | 495 |
| RPA-T4 | CD4 | None | 738 |
|  |  | AMD3100 (CXCR4) | 752 |
| 12G5 | CXCR4 | None | 642 |
|  |  | TAK779 (CCR5) | 655 |
|  |  | Compound A (CCR5) | 647 |
| 44717.111 | CXCR4 | None | 304 |
|  |  | CXCL12 (CXCR4) | 42 |

**^a^**The concentrations of the competing ligands were: AMD3100 (241 nM), TAK779 (2.4 μM), Compound A (2.4 μM) and CXCL12 (500 nM).

**^b^**The mean fluorescence intensities reported were derived from a single experiment. The experiment was repeated twice with comparable results.
